# The Fungal and Bacterial Rhizosphere Microbiome Associated With Grapevine Rootstock Genotypes in Mature and Young Vineyards

**DOI:** 10.3389/fmicb.2019.01142

**Published:** 2019-05-22

**Authors:** Carmen Berlanas, Mónica Berbegal, Georgina Elena, Meriem Laidani, José Félix Cibriain, Ana Sagües, David Gramaje

**Affiliations:** ^1^Instituto de Ciencias de la Vid y del Vino, Consejo Superior de Investigaciones Científicas – Universidad de la Rioja – Gobierno de La Rioja, Logroño, Spain; ^2^Instituto Agroforestal Mediterráneo, Universitat Politècnica de València, Valencia, Spain; ^3^EVENA, Sección de Viticultura y Enología del Gobierno de Navarra, Olite, Spain

**Keywords:** bacterial and fungal recruitment, black-foot disease, microbial ecology, microbiome, rhizosphere, rootstock selection

## Abstract

The microbiota colonizing the rhizosphere and the endorhizosphere contribute to plant growth, productivity, carbon sequestration, and phytoremediation. Several studies suggested that different plants types and even genotypes of the same plant species harbor partially different microbiomes. Here, we characterize the rhizosphere bacterial and fungal microbiota across five grapevine rootstock genotypes cultivated in the same soil at two vineyards and sampling dates over 2 years by 16S rRNA gene and ITS high-throughput amplicon sequencing. In addition, we use quantitative PCR (qPCR) approach to measure the relative abundance and dynamic changes of fungal pathogens associated with black-foot disease. The objectives were to (1) unravel the effects of rootstock genotype on microbial communities in the rhizosphere of grapevine and (2) to compare the relative abundances of sequence reads and DNA amount of black-foot disease pathogens. Host genetic control of the microbiome was evident in the rhizosphere of the mature vineyard. Microbiome composition also shifted as year of sampling, and fungal diversity varied with sampling moments. Linear discriminant analysis identified specific bacterial (i.e., *Bacillus*) and fungal (i.e., *Glomus*) taxa associated with grapevine rootstocks. Host genotype did not predict any summary metrics of rhizosphere α- and β-diversity in the young vineyard. Regarding black-foot associated pathogens, a significant correlation between sequencing reads and qPCR was observed. In conclusion, grapevine rootstock genotypes in the mature vineyard were associated with different rhizosphere microbiomes. The latter could also have been affected by age of the vineyard, soil properties or field management practices. A more comprehensive study is needed to decipher the cause of the rootstock microbiome selection and the mechanisms by which grapevines are able to shape their associated microbial community. Understanding the vast diversity of bacteria and fungi in the rhizosphere and the interactions between microbiota and grapevine will facilitate the development of future strategies for grapevine protection.

## Introduction

Plants have evolved to cope with biotic and abiotic stresses in association with soil microorganisms (Lemanceau et al., 2017). These microorganisms are known as plant microbiota and, together with the plant, they form an holobiont (Liu et al., 2018). Plant-soil microbiome interactions are complex and, until recent times, the study of these relationships has been mainly focused in the pathogenicity of some microbial agents and how they use and compete for the resources (Philippot et al., 2013; Zancarini et al., 2013; Gilbert et al., 2014; Sapkota et al., 2015). Recent investigations have shown that soil microbiota can directly and indirectly interact with the plants improving their fitness and health (Sapkota et al., 2015). For example, these interactions help plants to deal with abiotic stress and diseases, improving the exchange of substances such as nitrogen or phosphate, or by acting as biocontrol agents through competition with pathogens (Reinhold-Hurek et al., 2015; Vega-Avila et al., 2015; Gallart et al., 2018).

Roots are surrounded by a narrow zone of soil known as rhizosphere. This area, which is influenced by the roots, has a high microbial diversity and its community structure is expected to be different than the one found in the bulk soil (Reinhold-Hurek et al., 2015). The rhizosphere microbiome community composition is affected by different factors, such as ambient conditions, soil properties, and background microbial composition (Qiao et al., 2017). In addition, plants are able to shape their rhizosphere microbiome, as evidenced by the fact that different plant species host specific microbial communities when grown on the same soil (Aira et al., 2010; Berendsen et al., 2012; Bazghaleh et al., 2015).

As reviewed by Philippot et al. (2013), plant roots release a huge variety of carbon-containing compounds known as rhizodeposits (nutrients, exudates, border cells, and mucilage) which make the rhizosphere more nutritive than the bulk soil, which is mostly mesotrophic/oligotrophic, inducing therefore changes on soil microbial communities. It has been reported that the biodiversity in the rhizosphere is lower than in the corresponding bulk soil (Reinhold-Hurek et al., 2015; Lemanceau et al., 2017) since carbon availability often limits microbial growth (Dennis et al., 2010). Rhizodeposits released by the plants considerably vary according to the age and development of plants, among species and even among different genotypes of the same species (Inceoǧlu et al., 2010; Philippot et al., 2013; Gilbert et al., 2014; Bazghaleh et al., 2015; Hacquard, 2016; Wagner et al., 2016; Lemanceau et al., 2017; Qiao et al., 2017).

The rhizosphere is also the infection court where soil-borne pathogens establish a parasitic relationship with the plant. To infect root tissue, pathogens have to compete with members of the rhizosphere microbiome for available nutrients and microsites (Chapelle et al., 2016). Exploiting genetic variation in host plant species and understanding interactions between microbiota and their hosts plants will allow the rhizosphere microbiota to be incorporated into plant breeding programs to promote beneficial associations between plants and microorganisms.

Common grapevine (*Vitis vinifera* L.) is one of the most extensively grown and economically important woody perennial fruit crop worldwide with an annual production in 2014 exceeding 74 million tons of grapes and 30 million tons of wine (FAO, 2018). Since the late 19th century, *V. vinifera* cultivars have been grafted onto resistant rootstock of other *Vitis* species and hybrids to combat the devastating root phylloxera pest. Several major criteria have been outlined for choosing rootstocks: resistance to phylloxera and nematodes, and adaptability to drought, salinity, limestone content, and poor mineral nutrition (Reynolds and Wardle, 2001). In addition, the rootstock influence may affect scion vigor, yields, and fruit and wine qualities (Warschefsky et al., 2016).

Plant genetic control over microbial communities in the rhizosphere has been reported for different genotypes of the same species (Aira et al., 2010; Bouffaud et al., 2012; Peiffer et al., 2013; Marques et al., 2014; Jiang et al., 2017; Gallart et al., 2018). However, within grapevine species, the impact of genetic variation on the composition of the bacterial and fungal microbiota is poorly understood. In a recent study, Marasco et al. (2018) observed that five grapevine genotypes influenced the bacterial microbiome from both the root tissues and the rhizosphere fractions at a single vineyard, sampling date and year.

To better understand the players and processes that operate in the rhizosphere, a variety of molecular techniques, such as metagenomics have been applied over the past decade. Here, we characterize the rhizosphere bacterial and fungal microbiota across five grapevine rootstock genotypes cultivated in the same soil at two vineyards and sampling dates over 2 years by 16S rRNA gene and ITS high-throughput amplicon sequencing (HTAS). This design allowed us to evaluate the effect of the growing region, year, sampling date, grapevine genotype, and their interactions on the bacterial and fungal community diversity. In addition, we used quantitative Polymerase Chain Reaction (qPCR) approach to measure the relative abundance and dynamic changes of fungal pathogens associated with black-foot disease, one of the main soil-borne fungal diseases affecting grapevine production worldwide.

## Materials and Methods

### Sample Collection

Grapevine rhizosphere samples of five rootstocks (110 R, 140 Ru, 1103 P, 41 B, and 161-49 C) were collected at two vineyards located in Aldeanueva de Ebro (abbreviated as “Aldea”) (La Rioja, Spain) and Olite (Navarre, Spain). Features of the selected rootstocks are reported in Supplementary Table 1 (Martínez-Cutillas et al., 1990; Hidalgo, 2002; Keller, 2010). All the selected rootstocks were cultivated in the same vineyard and had been grafted onto Tempranillo cultivar. Soil physicochemical properties showed significant differences between soil types. Climate and soil management practices for fertilization, irrigation, and disease control also varied between vineyards (Supplementary Table 2). Aldea vineyard was 25-year-old vines at the moment of sampling and contained four randomized blocks of 48 vines per rootstock and block. Olite vineyard was 7-year-old vines at the moment of sampling and contained three randomized blocks of 15 vines per rootstock and block. In each vineyard, three rhizosphere samples were randomly collected per rootstock at two sampling dates (June and November) over 2 years (2016 and 2017). Sampled vines did not show any symptom of disease or nutrient deficiency. A total of 60 samples were collected per vineyard.

Rhizosphere soil samples were collected with a sterile spade close to the stem at depths of 40 to 50 cm, where the root system was denser. All samples were stored in sterile bags on dry ice at the time of sampling, and brought to the laboratory for further processing within 24 h from the time of sampling. The sampled roots with rhizosphere soil particles attached were placed in sterile tubes containing 9 mL of physiological solution (9 g/L NaCl). The tubes were vortexed for 5 min to detach the soil particles and then centrifuged at 4000 rpm for 5 min. The supernatant was discarded and the remaining soil fraction was used for DNA extraction.

### DNA Extraction and Sequencing

The rhizosphere DNA was extracted from 0.5 g sample using the DNeasy PowerSoil Kit (Qiagen, Hilden, Germany) and DNA samples were randomized across plates. The bacterial V4 region of the 16S rRNA gene was amplified using the protocol described by Lundberg et al. (2013). The universal primer pair 515F and 806R was used to generate bacterial-derived 16S rRNA amplicons. PNA PCR clamps were used to reduce host organelle contamination. The fungal ITS2 region was amplified using the universal primers ITS3/KYO2 and ITS4 (Toju et al., 2012). All primers were modified to include Illumina adapters^1^. Each 25 μl reaction contained 12.5 μl of HiFi HotStart Ready Mix (KAPA Biosystems, Woburn, MA, United States), 1.0 μl of each primer (10 μM), 2.5 μl of DNA template (5 ng/μl), and 8.0 μl PCR-grade water. PCR amplifications (performed in triplicate for each sample) consisted of a 3 min denaturation at 95°C; 25 cycles of 30 s at 95°C, 30 s at 55°C and 30 s at 72°C; and 5 min at 72°C. Samples were cleaned using the AMPure beads XP purification system (Beckman Coulter, United Kingdom) and sequenced on the Illumina MiSeq platform at the Fundación FISABIO (Valencia, Spain) facility using a 2 × 300 nucleotide paired reads protocol.

### Data Analysis

Raw forward and reverse reads for each sample were assembled into paired-end reads considering the minimum overlapping of 50 nucleotides and a maximum of one mismatch within the region using the fastq-join tool from the ea-tools suite (Aronesty, 2011). The paired reads were then quality trimmed with a minimum of Q20. Sequences without either primer were discarded. Chimeric sequences were identified and filtered using the Usearch tool (Edgar, 2010, 2018). The UClust algorithm (Edgar, 2013) in QIIME (Caporaso et al., 2010) was used to cluster sequences at a 97% sequence similarity against UNITE dynamic database (Abarenkov et al., 2010) for ITS reads and Greengenes database (DeSantis et al., 2006) using the QIIME implementation of the RDP classifier for 16S rRNA reads (Caporaso et al., 2010). A tree was constructed from a gap-filtered alignment using FastTree (Price et al., 2009). A final OTU table was created excluding unaligned sequences and singletons. OTUs with no kingdom-level classification or matching chloroplast, mitochondrial, or Viridiplantae sequences were then removed from the data set. Good’s coverage values were calculated using the Mothur computer software (Schloss et al., 2009). The rarefied OTU table and the phylogenetic tree were used as inputs for the subsequent analyses of α- and β- diversity. The OTU table was log transformed for statistical analysis (McMurdie and Holmes, 2014). As a final filter, taxa whose total abundances were less than 1% of the mean abundance were excluded, and only the OTUs present in at least two-thirds of the replicates of each sample were selected.

### Bacterial and Fungal Diversity, Taxonomy Distribution and Statistical Analysis

Biodiversity indexes and principle statistics analyses on taxonomic profiles were analyzed in R version 3.5 using the vegan (Oksanen et al., 2018) and Phyloseq packages (McMurdie and Holmes, 2014). Data in each vineyard was analyzed separately due to the differences in soil chemistry and climate (Supplementary Table 2). Technical noise (variation attributable to sequencing depth or batch effects) was controlled by including MiSeq run as a random effect.

Within sample type, α-diversity estimates were calculated by analyzing the Chao1 richness and Shannon diversity in Phyloseq package, as implemented in the tool MicrobiomeAnalyst (Dhariwal et al., 2017). The normalized OTU table was analyzed using Bray Curtis metrics (Bray and Curtis, 1957) and utilized to evaluate the β- diversity and to construct PCoA plots (Vázquez-Baeza et al., 2013) using MicrobiomeAnalyst. In order to compare bacterial and fungal communities composition and to partition of variance in different categories, Bray–Curtis distance matrices were subjected to PERMANOVA (Anderson, 2001) using the adonis function with a permutation number of 999 available in the vegan package of R. PERMANOVA was performed to investigate which OTUs significantly differed in abundance among experimental factors.

The variance-partitioning model tests for effects of year, sampling date and genotype on microbiome communities, while year-by-genotype and date-by-genotype interaction terms describe how the distinct fungal and bacterial communities at different common rootstocks respond differently to each of these factors. The linear mixed models were fit using the lme4 package (Bates et al., 2015). Statistical significance of fixed predictors (Year + Sampling Date + Genotype + Year × Genotype + Date × Genotype) was assessed using Type III ANOVA with Satterthwaite’s approximation of denominator degrees of freedom in the package InnerTest (Kuznetsova et al., 2016), and of random effects (MiSeq run) using likelihood ratio tests. This model was used to predict community descriptors that were continuous and approximately normally distributed in α-diversity metrics (Shannon entropy and Chao1 estimated richness) as described above.

The Linear Discriminant Analysis Effect Size (LEfSe) algorithm was used to identify taxa (genus level or higher) that differed in relative abundance between the rootstocks (Segata et al., 2011). The online Galaxy Version 1.0 interface (The Huttenhower Lab, 2018) was used, the threshold for the logarithmic LDA score was set at 1.0 and the Wilcoxon *p*-value at 0.05. The results are displayed in a cladogram and a bar graph. A Similarity Percentages (SIMPER) analysis was performed with PRIMER 6 software to explore the dissimilarities between the rootstock factor. Summarized taxa tables at the phylum and genera levels were used to investigate the phylogenetic groups that contribute to the dissimilarity. Unclassified OTUs amounting to less than 3% of the relative abundance in the rhizosphere were discarded from the analysis, according to Marasco et al. (2018). The bacterial and fungal OTUs shared among vineyards and rootstocks were defined by a Venn-diagram analysis using the software available at (Van de Peer et al., 2018).

### Quantitative PCR Amplification and Quantification of Black-Foot Disease Pathogens

Quantitative PCR analyses were performed with the DNA extracted from the soil samples, as Agustí-Brisach et al. (2014) developed in previous research, using the primers YT2F and Cyl-R (Dubrovsky and Fabritius, 2007; Tewoldemedhin et al., 2011). These primers amplify the main *Cylindrocarpon*-like asexual morphs associated with black-foot disease, in particular those belonging to the genera *Dactylonectria*, *Ilyonectria*, *Neonectria*, and *Thelonectria*. Rotor-Gene 6000 real-time rotary analyzer (Qiagen, Hilden, Germany) was used to perform the qPCR amplifications. Each reaction contained 2 μl of DNA, 1× of SYBR Premix Ex Taq II (Tli RNase H Plus) (Takara Bio Inc., Shiga, Japan) and 0.4 μM of each primer. The reaction mix was adjusted to a final volume of 20 μl with sterile distilled water. The thermocycling profile consisted of 30 s at 95°C and 50 cycles of 10 s at 95°C, 10 s at 60°C, and 30 s at 72°C. To evaluate amplification specificity, melting curve analysis was performed at the end of the qPCR runs according to the manufacturer’s recommendations. Each analysis included three replicates of each sample, a non-template control reaction (water) and a positive control containing DNA extracted from a pure culture of the *Dactylonectria torresensis* isolate GTMF DT097, obtained from the collection of the Instituto Agroforestal Mediterráneo, Universitat Politècnica de Valencia, Spain. *D. torresensis* is the most common fungal species associated with black-foot diseased vines in Italy (Carlucci et al., 2017), Portugal (Reis et al., 2013), and Spain (Berlanas et al., 2017). For DNA extraction, fungal mycelium of this isolate grown on potato dextrose agar (PDA, Biokar-Diagnostics, Zac de Ther, France) for 2 weeks at 25°C in darkness, was scraped from the surface of the plate with a sterile scalpel. Total DNA was extracted using the E.Z.N.A. Plant Miniprep kit (Omega Bio-Tek, Doraville, United States) following the manufacturer’s instructions and mycelia was previously homogenized with 4 steel beads of 2.38 mm and 2 of 3 mm diameter (Qiagen, Hilden, Germany) using a FastPrep-24TM5G (MP Biomedicals, California, United States) at 5 m/s for 20 s twice. DNA extracted was quantified with Invitrogen Qubit 4 Fluorometer (Thermo Fisher Scientific, Waltham, United States).

DNA of the *Cylindrocarpon*-like asexual morphs species was quantified using a standard curve constructed with the isolate GTMF DT097, consisting of a dilution series from 275 μg/μL to 0.275 fg/μL. Quantitative PCR analysis were perform as previously explained and the standard curve was generated following the MIQE guidelines (Bustin et al., 2009), by plotting quantification cycle (C_q_) values obtained for each specific DNA concentration, versus the logarithm of the initial concentration of isolate DNA. The mean DNA concentration and the standard deviation were determined from three replicates per dilution. Sensitivity of the qPCR assay was assessed using the standard curve to determine the minimum DNA concentration that can be detected. The amplification efficiency (E) and the coefficient of determination (*R*^2^) of the standard curve were obtained using the Rotor-Gene 6000 Series software v. 1.7 (Qiagen, Hilden, Germany). Signal threshold levels were set automatically by the instrument software and the limit of detection (LOD) was identified by the last dilution when successful qPCR amplification of DNA occurred, accompanied by a melting curve peak temperature specific to *D. torresensis*.

Values from the *Cylindrocarpon*-like asexual morphs number of OTUs and DNA concentration were transformed by log (n/N ∗ 1000 + 1). Where n was the number of OTUs or the DNA concentration detected on each sample and N was the total number of OTUs and the total DNA concentration detected. An analysis of correlation between both transformed datasets was performed in R version 3.5 using the corrr package.

## Results

### High-Throughput Amplicon Sequencing

After paired-end alignments, quality filtering and deletion of chimeric, singletons, and mitochondrial and chloroplast sequences, a total of 4,337,395 bacterial 16S rRNA sequences and 6,216,366 fungal internal transcribed spacer (ITS) sequences were generated from 117 (three samples were removed from the analysis due to the low number of sequence reads) and 120 samples, respectively, and assigned to 975 bacterial and 567 fungal operational taxonomic units (OTUs) (Supplementary Table 3). Good’s coverage values indicated that on average 94.5 and 90.1% of the total species richness were accounted for in bacteria and fungal communities, respectively (Supplementary Table 4). Chao1 diversity estimator ranged from 143.6 to 549.5 in the bacterial microbiome, and from 90.5 to 254.9 in the fungal microbiome. Shannon diversity estimator ranged from 1.80 to 4.68 in the bacterial microbiome, and from 1.80 to 3.84 in the fungal microbiome (Supplementary Table 4).

### Core Grapevine Phylogeny Between Vineyards

**FIGURE 1:**
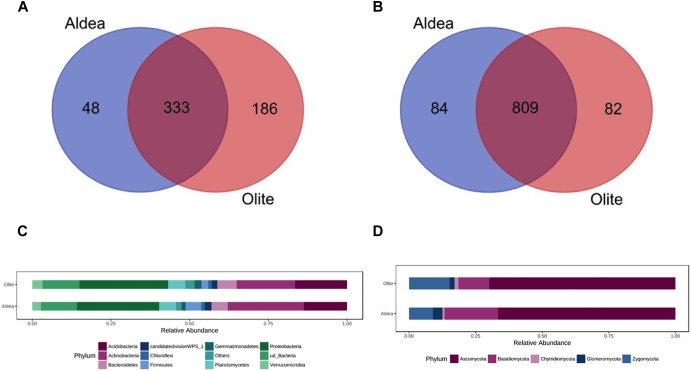
Venn diagram illustrating the overlap of the OTUs identified in the bacterial **(A)** and fungal **(B)** microbiota between vineyards. Relative abundance of different bacterial **(C)** and fungal **(D)** phyla in the rootstock rhizospheres in both vineyards representing OTUs showing more than 1% relative abundance of all reads and present in at least 2/3 of replicates. Phyla representing less than 1% of the total reads are grouped in “Others”.

The two habitats used as vineyard sites (Aldeanueva del Ebro, abbreviated “Aldea” in the figures and tables; and Olite) were separated by 45 km, and varied in most of soil physicochemical properties (Supplementary Table 2). Bacterial communities of rhizosphere soil samples did not differ significantly between vineyards (Supplementary Table 5). However, α-diversity differed among sites when studying the fungal microbiota, and principal coordinates analysis (PCoA) of Bray Curtis data demonstrated that vineyard was the primary source of β-diversity (Supplementary Figure 1). Comparing the fungal and bacterial microbiota of the two vineyards, 82.9 and 58.7% of bacterial and fungal OTUs, respectively, were shared between vineyards, demonstrating the existence of a “core” grape phylogeny that is independent of the growing region (Figure 1).

The relative abundance of bacterial and fungal phyla detected across all samples is shown in Figure 1. In both vineyards, the bacterial phyla Proteobacteria (26.1 and 28.1% in Aldea and Olite, respectively) and Actinobacteria (24.1 and 18.5%) represented almost 50% of the total bacteria detected. These phyla were followed by Acidobacteria (13.7 and 16.4%), unidentified bacteria (11.4 and 11.7%), and Bacteroidetes (5.2 and 6.1%) (Figure 1). The most abundant families within the Proteobacteria phylum were unidentified families from the order Rhizobiales (13.0 and 10.4% in Aldea and Olite, respectively), unidentified families from the class Betaproteobacteria (9.8 and 13.0%) and Sphingomonadaceae (7.6 and 10.7%). The most abundant families within the Actinobacteria phylum were unidentified Actinobacteria (29.1 and 22.5% in Aldea and Olite, respectively), Gaiellaceae (16.0 and 15.2%) and Streptomycetaceae (6.2 and 6.7%) (Supplementary Figure 2). Regarding the fungal taxa, the most abundant fungal phylum was Ascomycota (66.6 and 69.9% in Aldea and Olite, respectively), followed by Basidiomycota (20.1 and 11.5%) and Zygomycota (8.9 and 15.2%) (Figure 1). The most abundant families within the Ascomycota phylum were Nectriaceae (15.4%), unidentified Ascomycota (8.8%), and Bionectriaceae (9.1%) in Aldea vineyard, and Nectriaceae (17.7%), unidentified Ascomycota (11.1%), Pyronemataceae (9.6%), and Trichocomaceae (8.4%) in Olite vineyard (Supplementary Figure 2).

### Host Genetic Influence on the Rhizosphere Microbiota

**FIGURE 2:**
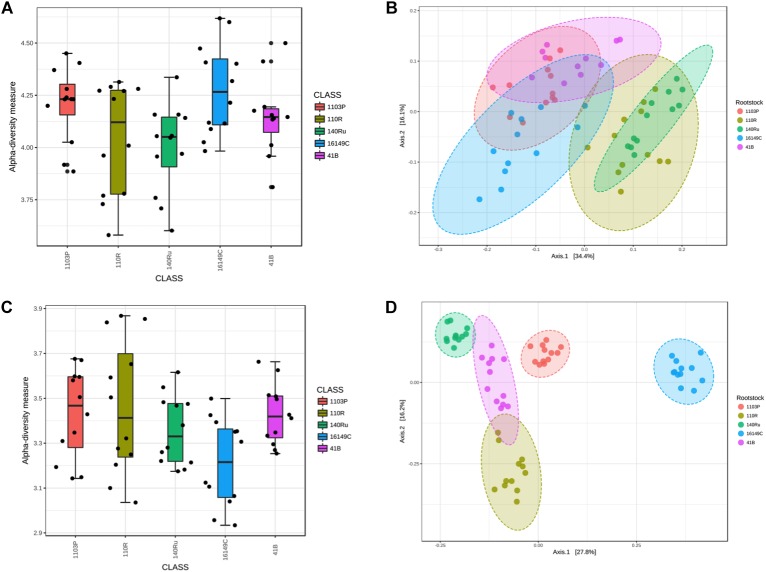
Box plot illustrating the differences in Shannon diversity measures of the bacterial **(A)** and fungal **(C)** communities in the grapevine rootstocks in Aldea vineyard. Principal Coordinate Analysis (PCoA) based on Bray Curtis dissimilarity metrics, showing the distance in the bacterial **(B)** and fungal **(D)** communities among grapevine rootstocks.

Bacterial and fungal diversity in rhizosphere soil samples differed significantly among rootstocks in Aldea vineyard. However, plant genotype did not predict Chao1 diversity (Table 1). Host genotype was the most important factor in structuring bacterial (*R*^2^ = 0.65, *P* < 0.001) and fungal (*R*^2^ = 0.86, *P* < 0.001) communities in the entire dataset, and also when the data were split by year and date (Table 2). A PCoA further demonstrated the variation in the total dataset could be attributed to host genotype in Aldea vineyard (Figure 2). In Olite vineyard, plant genotype had a much weaker influence on rhizosphere-associated bacterial and fungal communities. Host genotype did not predict any summary metrics of rhizosphere α and β-diversities (Tables 1, 2).

**Table 1:** Experimental factors predicting α-diversity of rhizosphere associated fungal and bacterial communities in Aldea and Olite vineyards.

Bacteria	Aldea		Olite	
	Shannon	Chao1	Shannon	Chao1
Genotype	*F*_4,54_ = 3.47 ***P* = 0.0134**	*F*_4,54_ = 0.34 *P* = 0.8480	*F*_4,54_ = 0.90 *P* = 0.4693	*F*_4,54_ = 0.32 *P* = 0.8648
Year	*F*_1,57_ = 6.83 ***P* = 7.3e-09**	*F*_1,57_ = 17.39 ***P* = 1.5e-20**	*F*_1,57_ = 4.66 ***P* = 1.6e-04**	*F*_1,57_ = 7.55 ***P* = 4.7e-10**
Year × Genotype	*F*_4,49_ = 0.73 ***P* = 0.0122**	*F*_4,49_ = 1.48 *P* = 0.3661	*F*_4,49_ = 2.33 *P* = 0.0623	*F*_4,49_ = 6.08 *P* = 0.2143
Date	*F*_1,57_ = 0.05	*F*_1,57_ = 0.18	*F*_1,57_ = 0.68	*F*_1,57_ = 0.13
	*P* = 0.9555	*P* = 0.8502	*P* = 0.4989	*P* = 0.8941
Date × Genotype	*F*_ 4,49_ = 1.55	*F*_4,49_ = 0.74	*F*_4,49_ = 0.19	*F*_4,49_ = 1.67
	*P* = 0.1812	*P* = 0.7702	*P* = 0.1802	*P* = 0.2561
MiSeq run	*χ*^2^_1_ = 0.55 *P* = 0.3623	*χ*^2^_1_ = 0.74 *P* = 0.4565	*χ*^2^_1_ = 0.28 *P* = 0.7712	*χ*^2^_1_ = 1.59 *P* = 0.3421
**Fungi**				
Genotype	*F*_4,55_ = 2.80 ***P* = 0.0232**	*F*_4,55_ = 1.12 *P* = 0.3529	*F*_4,55_ = 0.82 *P* = 0.5130	*F*_4,55_ = 2.27 *P* = 0.0929
Year	*F*_1,58_ = 0.95 *P* = 0.3415	*F*_1,58_ = 10.62 ***P* = 3.2e-15**	*F*_1,58_ = 0.37 *P* = 0.7112	*F*_1,58_ = 5.25 ***P* = 3.5e-06**
Year × Genotype	*F*_4,50_ = 2.85 *P* = 0.1126	*F*_4,50_ = 1.15 *P* = 0.3601	*F*_4,50_ = 0.35 *P* = 0.1831	*F*_4,50_ = 3.85 *P* = 0.3126
Date	*F*_1,58_ = 8.52	*F*_1,58_ = 2.17	*F*_1,58_ = 0.44	*F*_1,58_ = 1.31
	***P* = 1.08e-11**	*P* = 0.0640	*P* = 0.6597	*P* = 0.1937
Date × Genotype	*F*_ 4,50_ = 0.71	*F*_4,50_ = 0.91	*F*_4,50_ = 1.91	*F*_4,50_ = 6.81
	***P* = 0.0112**	*P* = 0.2903	*P* = 0.6351	*P* = 0.7443
MiSeq run	*χ*^2^_1_ = 0.74	*χ*^2^_1_ = 2.92	*χ*^2^_1_ = 1.77	*χ*^2^_1_ = 0.12
	*P* = 0.4912	*P* = 0.2551	*P* = 0.8135	*P* = 0.7331


*ANOVA, analysis of variance. Statistics describe linear random-intercept models of Shannon diversity and Chao1 richness in the rhizosphere. All *P*-values were corrected for multiple comparisons using the sequential Bonferroni correction. Significance was assessed using Type III ANOVA with *F*-tests for fixed effects and likelihood ratio tests for the random effect. Bold values indicate statistically significant results after correction for multiple comparisons. *P* < 0.05.*

**Table 2:** Adonis test of category effect on bacterial and fungal Bray–Curtis distance matrix.

Bacteria	Aldea			Olite		
Dataset	Factor	*R*^2^	*P*-value	Factor	*R*^2^	*P*-value
Total	Genotype	0.658	0.001	Genotype	0.058	0.015
	Year	0.163	0.001	Year	0.494	0.001
	Date	0.109	0.002	Date	0.059	0.004
110 R	Year	0.564	0.002	Year	0.438	0.005
	Date	0.028	0.116	Date	0.204	0.066
140 Ru	Year	0.235	0.006	Year	0.458	0.005
	Date	0.355	0.002	Date	0.092	0.333
1103 P	Year	0.220	0.011	Year	0.379	0.005
	Date	0.461	0.002	Date	0.174	0.036
41 B	Year	0.087	0.071	Year	0.453	0.005
	Date	0.670	0.002	Date	0.129	0.092
161 49 C	Year	0.228	0.003	Year	0.471	0.005
	Date	0.228	0.005	Date	0.221	0.040
2016	Genotype	0.868	0.001	Genotype	0.206	0.031
	Date	0.067	0.035	Date	0.165	0.001
2017	Genotype	0.768	0.001	Genotype	0.240	0.001
	Date	0.135	0.004	Date	0.138	0.002
June	Genotype	0.634	0.001	Genotype	0.145	0.365
	Year	0.110	0.005	Year	0.331	0.001
November	Genotype	0.831	0.001	Genotype	0.240	0.020
	Year	0.123	0.004	Year	0.354	0.001
**Fungi**						
Total	Genotype	0.864	0.001	Genotype	0.096	0.027
	Year	0.052	0.004	Year	0.564	0.001
	Date	0.084	0.001	Date	0.042	0.005
110 R	Year	0.183	0.122	Year	0.438	0.005
	Date	0.501	0.002	Date	0.204	0.066
140 Ru	Year	0.142	0.137	Year	0.458	0.005
	Date	0.615	0.002	Date	0.092	0.333
1103 P	Year	0.266	0.031	Year	0.379	0.005
	Date	0.496	0.002	Date	0.174	0.036
41 B	Year	0.241	0.033	Year	0.453	0.005
	Date	0.425	0.002	Date	0.129	0.092
161 49 C	Year	0.191	0.066	Year	0.471	0.005
	Date	0.472	0.002	Date	0.221	0.040
2016	Genotype	0.841	0.001	Genotype	0.144	0.305
	Date	0.110	0.002	Date	0.070	0.002
2017	Genotype	0.928	0.001	Genotype	0.274	0.001
	Date	0.130	0.002	Date	0.127	0.002
June	Genotype	0.808	0.001	Genotype	0.220	0.012
	Year	0.066	0.080	Year	0.289	0.001
November	Genotype	0.753	0.001	Genotype	0.200	0.003
	Year	0.105	0.004	Year	0.208	0.001


**FIGURE 3:**
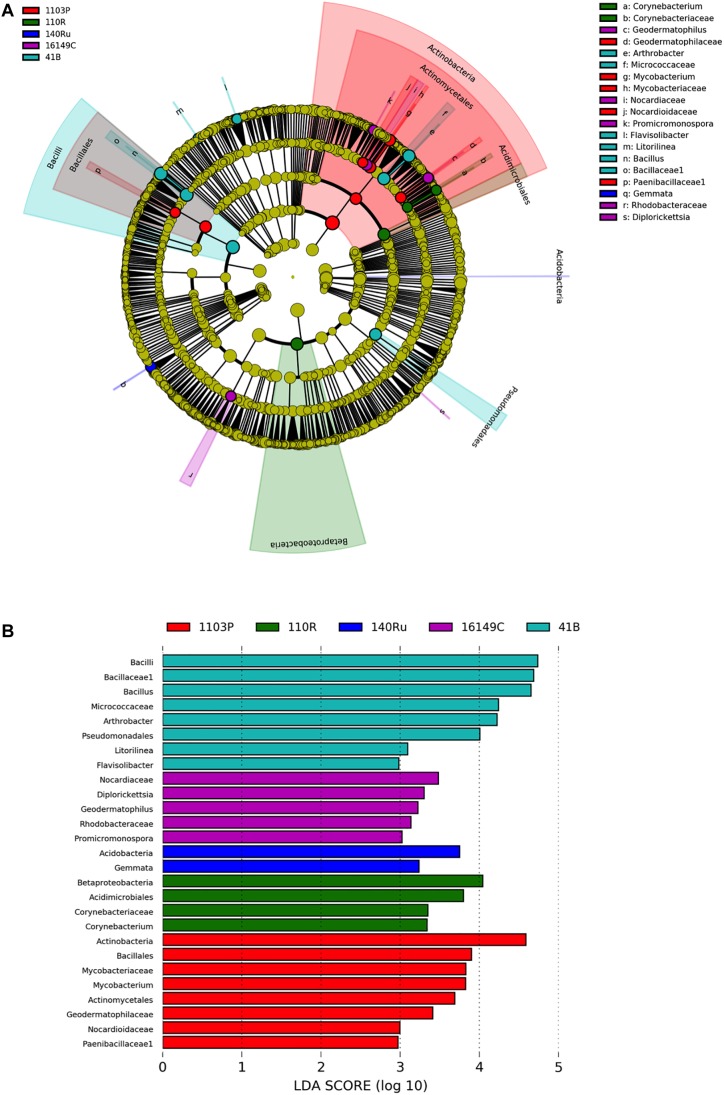
LEfSe was used to identify the most differentially abundant taxa among grapevine rootstocks in Aldea vineyard. Cladogram generated by LEfSe indicating differences of bacteria **(A)** at phylum, class, family, and genus levels between the five groups (relative abundance ≤0.5%). Each successive circle represents a phylogenetic level. Color regions indicate taxa enriched in the different rootstocks. Differing taxa are listed on the right side of the cladogram. Bar graph showing LDA scores for bacteria **(B)**. Only taxa meeting an LDA significant threshold >2 are shown.

**FIGURE 4:**
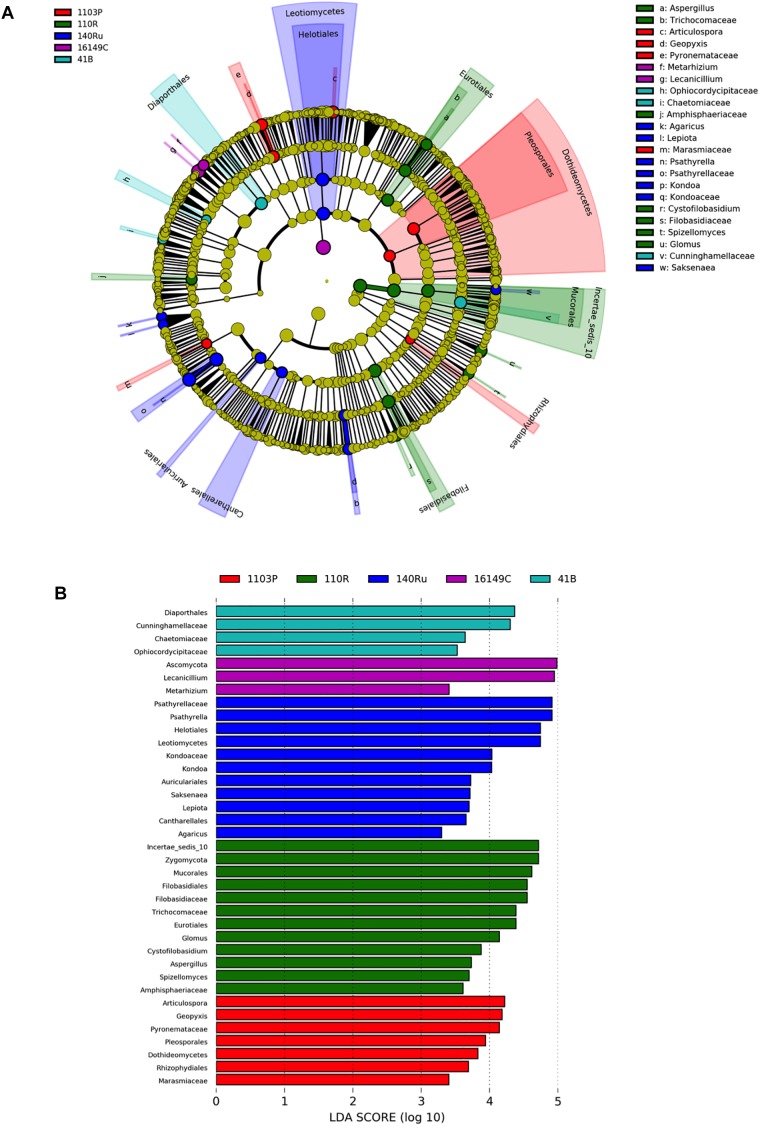
LEfSe was used to identify the most differentially abundant taxa among grapevine rootstocks in Aldea vineyard. Cladogram generated by LEfSe indicating differences of fungi **(A)** at phylum, class, family, and genus levels between the five groups (relative abundance ≤0.5%). Each successive circle represents a phylogenetic level. Color regions indicate taxa enriched in the different rootstocks. Differing taxa are listed on the right side of the cladogram. Bar graph showing LDA scores for fungi **(B)**. Only taxa meeting an LDA significant threshold >2 are shown.

The linear discriminant analysis effect size (LEfSe) detected 27 bacterial and 36 fungal clades in the rhizospheres, which discriminated the microbial communities between the different rootstock genotypes in Aldea vineyard (Figure 3, 4). Both rootstocks 1103 P and 41 B showed higher number of differentially abundant bacterial clades (8 each) than the other rootstocks (5, 4, and 2 in 161-49 C, 110 R, and 140 Ru, respectively). The dominant bacterial phyla were Firmicutes (37%) in rootstock 41B, Actinobacteria and Planctomycetes (50% each) in rootstock 140 Ru, and Actinobacteria in rootstocks 161-49 C, 110 R, and 1103 P (60, 75, and 75%, respectively) (Figure 3). The dominant fungal phyla were Basidiomycota (73%) in rootstock 140 Ru, and Ascomycota in rootstocks 41 B, 161-49 C, 110 R, and 1103 P (75, 100, 36, and 71%, respectively) (Figure 4).

The rootstock-pairs dissimilarity, due to phyla and genera contribution in the rhizosphere was calculated by SIMPER (similarity percentages) analysis (Supplementary Table 6). Higher microbiome dissimilarity among rootstocks was revealed in Aldea vineyard compared to Olite vineyard, considering bacterial (Supplementary Table 6A) and fungal phyla (Supplementary Table 6C), and bacterial (Supplementary Table 6B) and fungal genera (Supplementary Table 6D) distribution. Firmicutes and Acidobacteria were the major phyla that contribute to differentiate the bacterial communities associated with the different rootstock types in Aldea and Olite vineyards, respectively (Supplementary Table 6A). Several genera were predominant and determined the dissimilarities among rootstocks such as *Bacillus* in Aldea vineyard or *Aridibacter* in Olite vineyard. The genus *Bacillus* appeared to be rhizosphere genotype biomarker of 140 Ru and 161-49 C rootstocks (Supplementary Table 6B). The fungal phyla Ascomycota and Basidiomycota contributed to the dissimilarity among rootstocks in Aldea vineyard, while only the phylum Basidiomycota contributed to differentiate fungal communities among rootstocks (Supplementary Table 6C). The fungal genera *Geopyxis*, *Clonostachys*, and *Lecanicillium* determined the dissimilarities among rootstocks in Aldea vineyard, being *Geopyxis* a rhizosphere genotype biomarker of 110 R rootstock and *Clonostachys* of 1103 P and 140 Ru rootstocks (Supplementary Table 6D). In Aldea vineyard, 161-49 C rootstock showed the highest dissimilarity with the other rootstocks in bacterial and fungal microbiome distribution.

### Year Strongly Influenced Microbiomes

Our results demonstrate that bacterial microbiome varied profoundly between years. This pattern was consistent to community-level measure of α- diversity in both Aldea and Olite vineyards (Table 1) Richness increased between 2016 and 2017 in both vineyards (Supplementary Figure 3). However, year of sampling affected the Bray Curtis metric of β-diversity in only Olite vineyard (*R*^2^ = 0.494) (Supplementary Figure 3). Regarding the fungal microbiome, richness also varied between vineyards and increased between 2016 and 2017 in both vineyards (Table 1 and Supplementary Figure 4). However, year of sampling did not predict Shannon diversity and affected the Bray Curtis metric of β-diversity in only Olite vineyard (Table 2 and Supplementary Figure 4). Sampling date also contributed to α-diversity variation indicating temporal changes in relative abundance of fungal OTUs in Aldea vineyard. Fungal composition decreased between June and November (Table 1 and Supplementary Figure 5). Fungal community structure varied individually in each rootstock with date (*R*^2^ ranging from 0.42 to 0.61), but not in the total dataset (*R*^2^ < 0.1) (Table 2).

### Rootstock-Specific and Shared Bacterial and Fungal Assemblages

**FIGURE 5:**
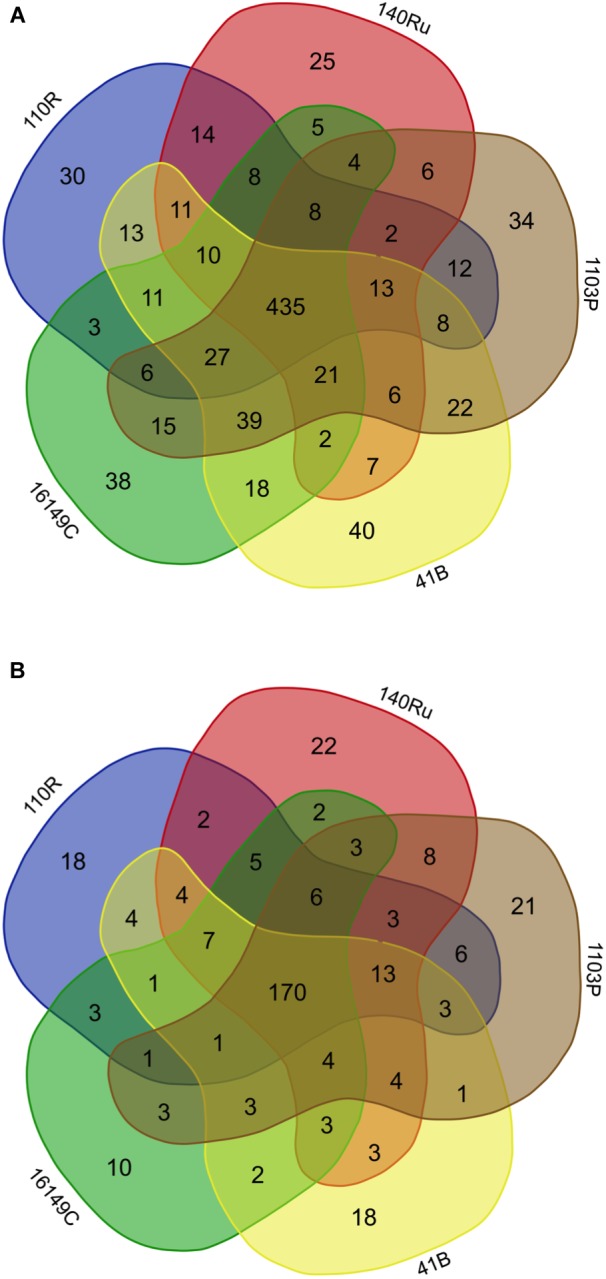
Venn diagrams showing the common and exclusive bacterial **(A)** and fungal **(B)** OTUs of the rhizosphere of the grapevine rootstocks in Aldea vineyard.

**FIGURE 6:**
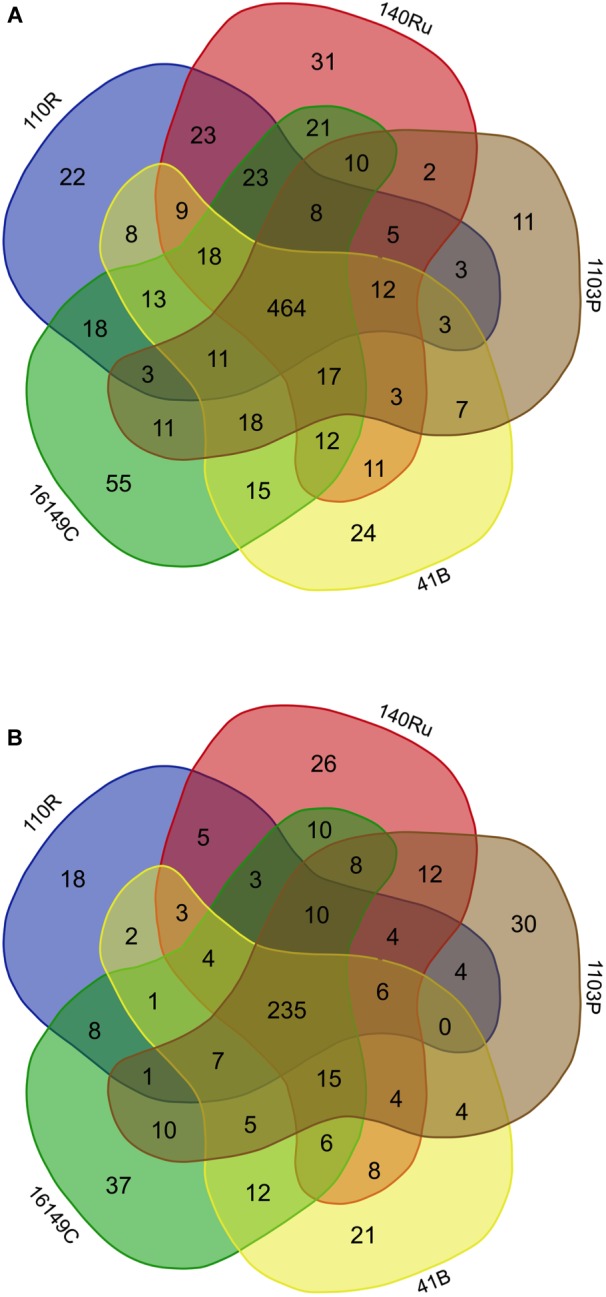
Venn diagrams showing the common and exclusive bacterial **(A)** and fungal **(B)** OTUs of the rhizosphere of the grapevine rootstocks in Olite vineyard.

The rhizosphere compartments of grapevine rootstocks showed specific fungal and bacterial OTUs for each rootstocks and a cluster of shared OTUs. In Aldea, specific OTUs associated with most of the rootstocks ranged from 4.3 to 5.8% of their bacterial communities (Figure 5). Specific OTUs associated with the rootstocks 140 Ru, 1103 P, 41 B and 110 R represented less than 9% of their fungal communities, where the 161-49C-specific OTUs enriched only 4.5% of the relative abundance (Figure 5). In Olite, specific OTUs associated with most of the rootstocks represented less than 9% of their bacterial and fungal communities, with the exception of bacterial communities associated with 140 Ru rootstock that represented 21.3% of its total (Figure 6). The OTUs that were unique in each of the grapevine rootstock are shown in Supplementary Tables 7, 8.

### Quantification of Black-Foot Disease Pathogens Using Quantitative PCR

The standard curve, constructed with serial dilutions of the DNA of *D. torresensis* isolate GTMF DT097, revealed high correlations between C_q_ and DNA, with *R*^2^-value of 0.99 and reaction efficiency of 0.90. The minimum DNA concentration detectable of *D. torresensis* was at C_q_ value of the dilution D7 thus, the limit of detection (LOD) was established at 2.75 fg/μL.

DNA of *Cylindrocarpon*-like asexual morphs was detected in all rootstock rhizosphere samples, in both vineyards and years, with concentrations ranging from 0.39 pg/μL to 4.06 pg/μL in Aldea 2016, from 3.52 pg/μL to 14.14 pg/μL in Aldea 2017, from 0.88 pg/μL to 8.45 pg/μL in Olite 2016 and from 2.65 pg/μL to 59 pg/μL in Olite 2017. The year and vineyard factors had a significant effect on *Cylindrocarpon*-like asexual morphs DNA concentration detected (*P* < 0.01). The concentration of DNA detected was significantly higher in Olite vineyard compared with Aldea vineyard, especially in year 2017. The rootstock factor had a significant effect on the DNA concentration detected in Aldea vineyard for 2017 samples (*P* = 0.0156). Rootstocks 161-49 C, 140 Ru, 1103 P, and 110R showed similar DNA concentrations values that were significantly lower when compared with 41 B rootstocks (Supplementary Figure 6). The analysis showed a positive significant correlation between the number of OTUs and the *Cylindrocarpon*-like asexual morphs DNA quantified using the real-time approach (*P* < 0.01, Spearman correlation coefficient = 0.72) (Supplementary Figure 7).

## Discussion

In this study, we characterized the rhizosphere microbial community composition across five commercial grapevine rootstock genotypes cultivated in the same soil at two vineyards and sampling dates over 2 years. The analysis of bacterial and fungal populations in the grapevine rhizosphere targeting 16S rRNA and ITS region, respectively, have been proved effective in previous studies (Corneo et al., 2014; Holland et al., 2016; Longa et al., 2017; Manici et al., 2017; Stefanini and Cavalieri, 2018). Especially for bacterial barcoding, the choice of partial sequence regions is pivotal and can significantly affect the results because the 16S rRNA gene regions have different divergence (Youssef et al., 2009). In our study, we used the V4 region because according to recent *in silico* studies (Youssef et al., 2009), V4 along with V5-V6, and V6-V7 regions were considered as the most suitable regions for metagenomic purposes because they provided estimates comparable to those obtained with the complete 16S rRNA gene sequence (Youssef et al., 2009).

Our study represents the first approach to investigate the rhizosphere fungal microbiome of grapevine by HTAS. In grapevine, the ecology of fungal communities is so far largely derived from the studies using pyrosequencing approach in bulk soil (Holland et al., 2016; Castañeda and Barbosa, 2017; Longa et al., 2017) or ARISA fingerprinting (Likar et al., 2017) and PCR-DGGE (Manici et al., 2017) approaches in rhizosphere soil. Even though the ITS region was ratified by The Fungal Barcoding Consortium (Schoch et al., 2012) as the universal DNA barcode for the fungal kingdom using the same gene section proposed by White et al. (1990), some recent reports point out its limitations for specific taxa. This region does not work well with taxa having narrow or no barcode gaps in their ITS regions, such as *Fusarium* or *Trichoderma* (Schoch et al., 2012). In addition, the correct identification of morphologically similar cryptic species using the ITS regions is still problematic due to the lack of consensus in the lineage-specific cut-off value for species determination (Nilsson et al., 2008).

The bacterial microbiomes of the different rootstocks were largely composed of Proteobacteria and Actinobacteria that accounted for almost 50% of the relative abundance in both vineyards. The predominant bacterial phyla found in this work is consistent with the results obtained in other studies in vineyard soil (Opsi et al., 2014; Vega-Avila et al., 2015; Castañeda and Barbosa, 2017; Longa et al., 2017; Marasco et al., 2018). Proteobacteria and Actinobacteria are known for their role in the carbon biochemical cycle and their production of second metabolites (Jenkins et al., 2009). The major fungal phyla detected in our study were largely composed of Ascomycota and Basiodiomycota that accounted for almost 75% of the relative abundance in both vineyards. Previous studies also agree on the most common fungal phyla detected in grapevines fields (Castañeda and Barbosa, 2017; Longa et al., 2017; Manici et al., 2017). These results suggest that vineyard microbiome in Navarre and La Rioja regions is partially conserved.

The results obtained in the Aldea vineyard showed a significant fraction of variation in fungal and bacterial diversity (both the α- and β-diversity) that could be attributed to host genetics. Recent research indicated that rootstock genotypes could have a notable influence in shaping the bacteria taxa distribution in the root and rhizosphere systems of grapevine (Marasco et al., 2018). This effect of the host genotype in the rhizosphere microbiome has been reported in other woody crops, such as apple (Liu et al., 2018) and pines (Gallart et al., 2018), as well as in several annual crops, such as maize (Peiffer et al., 2013), potato (Inceoǧlu et al., 2010), and chickpea (Bazghaleh et al., 2015). This could be due to the influence of the genotype in the root metabolism, including immune response and exudate composition, which impact in the rhizosphere microbiome (Wagner et al., 2016). Rootstocks show different level of tolerance to distinct diseases; and this could be decisive in their effect in the microbiome (Sapkota et al., 2015). Moreover, as reviewed by Liu et al. (2018), several studies hint to a possible co-evolution of the holobiont. However, further research is needed to validate this hypothesis. On the other hand, the Olite vineyard showed a lower microbiome dissimilarity among rootstocks, suggesting that the effect of genotype in shaping the microbiome might be influenced by other factors.

The differences between Olite and Aldea vineyards could lie in the soil physicochemical properties, in the soil and cultivar management practices, or in the age of the plants, being vines cultivated in Olite vineyard younger than in Aldea vineyard. Environmental heterogeneity, such as the soil physicochemical properties and moisture content have been identified as major factors shaping the spatial scaling of the rhizosphere microbiome in many previous studies (Costa et al., 2006; Tan et al., 2013; Schreiter et al., 2014), including grapevine (Fernández-Calviño et al., 2010; Corneo et al., 2014; Burns et al., 2015; Zarraonaindia et al., 2015; Holland et al., 2016). Soil physicochemical properties can also influence the population structure of specific soil-borne pathogens. For instance, Berlanas et al. (2017) observed that excessive calcium carbonate in soil may increase black-foot disease inoculum density.

Field management practices have been also reported as an important driver of the microbiome diversity (Santhanam et al., 2015; Sapkota et al., 2015; Hacquard, 2016; Gallart et al., 2018), including the grapevine soil microbiome (Vega-Avila et al., 2015; Likar et al., 2017; Longa et al., 2017). Nevertheless, other studies showed a long-term effect of cultivation rather than field management on soil microbial diversity (Buckley and Schmidt, 2001; Peiffer et al., 2013). Microbiome studies should consider the high degree of temporal variability in the sample design, because sampling the same point in different times can give different results due the variability of the own microbial community through time (Redford and Fierer, 2009). The year to year variation found in our study could be explained by the different root response to distinct environmental factors, such as temperature or precipitation (Wagner et al., 2016). Further research is needed to determine if environment plays a much greater role than host genetics in determining the composition of the rhizosphere microbiome of grapevine.

Several studies have remarked the effect of the growth stage of the plant in its associated rhizosphere microbiome (Baudoin et al., 2002; Inceoǧlu et al., 2010; Li et al., 2014; Okubo et al., 2014; Yuan et al., 2015; Wagner et al., 2016; Qiao et al., 2017). Changes in the quantity and quality of root exudates as plants develop have been proposed as the main source of variation of the rhizosphere microbiome composition present during different developmental stages of maize cultivars (Baudoin et al., 2002). However, most of the published studies are focused in annual plant systems. In grapevine, Manici et al. (2017) recently investigated shifts in bacterial and fungal communities between mature and young replaced vines in Italy. At a single sampling moment, these researchers concluded that long-term growth legacy overcame plant age in shaping rhizosphere microbiome (Manici et al., 2017). Further research is therefore needed to determine the long-term effect of the grapevine age on the associated microbiome as plants develop. This could be accomplished by comparing the rhizosphere microbiome (i) in a single vineyard over time, or (ii) in two vineyards in close proximity with identical environmental conditions and soils, but with vines on different aging process.

Our results showed that the root system type is able to select specific bacterial and fungal OTUs as biomarkers for the different genotypes. Members of the bacterial genus *Bacillus*, which was only found in 140 Ru and 161-49 C rootstocks in Aldea vineyard, has wide diversity of physiological ability with respect to heat, pH, and salinity. Therefore, *Bacillus* species can be found in a wide range of habitats, being a few of them pathogenic to vertebrates or invertebrates (Holt et al., 1994). *Bacillus subtilis* and *Bacillus amyloliquefaciens* have been described as potential biocontrol agents against *Aspergillus parasiticus* and stem rot disease (Le et al., 2018; Siahmoshteh et al., 2018). *In vitro* assays of the heat stable metabolites of *B. subtilis* showed promising results in reducing the growth of the fungal trunk pathogens *Lasiodiplodia theobromae*, *Phaeomoniella chlamydospora*, and *Phaeoacremonium minimum* (Alfonzo et al., 2009). Rezgui et al. (2016) recently identified several *B. subtilis* strains inhabiting the wood tissues of mature grapevines in Tunisia with antagonistic traits against fungal trunk pathogens. On the other hand, some species of the arbuscular mycorrhizal (AM) fungal genus *Glomus*, one of the most differentially abundant taxa for 110 R rootstock in Aldea vineyard, are cataloged as biocontrol agents (Tahat et al., 2010). For instance, inoculation of grapevine roots with *Rhizophagus irregularis* (syn. *Glomus intraradices*) reduced both the disease severity and the number of root lesions caused by black-foot disease pathogens (Petit and Gubler, 2006). AM fungi form one of the most interesting beneficial plant–micro-organism associations (Smith and Read, 2008) and are known to colonize the roots of the majority of land plants, including grapevines (Schreiner and Mihara, 2009; Trouvelot et al., 2015). Several genera within the Glomeromycota phylum have been identified from the rhizosphere samples obtainted in this study, namely *Claroideoglomus*, *Diversispora*, *Entrophosphora*, and *Rhizophagus*. Trouvelot et al. (2015) reported that soil management can greatly impact the diversity of AM fungi. In fact, AM fungal communities are highly influenced by the soil characteristics but also to a smaller extent by the host plant development stage (Schreiner and Mihara, 2009; Balestrini et al., 2010).

High-throughput amplicon sequencing is a powerful method for the analysis of microbial populations. It is accomplished by sequencing specific marker genes amplified directly from environmental DNA without prior enrichment or cultivation of the target population (Franzosa et al., 2015). The advantages of this approach is the detection of rare taxa at the genus level given the availability of large and comprehensive reference databases as well as several pipelines for bioinformatics analysis (Stefanini and Cavalieri, 2018). Drawbacks of HTAS include the biased relative quantification of bacterial communities since bacterial species bear various number of copies of 16S rRNA genes, the sequencing of matrix (e.g., grape ITS, chloroplast 16S) and the low confidence for taxonomic assignment at the species level (Stefanini and Cavalieri, 2018). A step forward consists of the understanding of how changes in the composition of microbial communities impact the population’s biological functions (Ravin et al., 2015). Unfortunately, HTAS only allows inference of functional annotation while in whole-genome sequencing, functional annotation can be carried out by gene enrichment (Stefanini and Cavalieri, 2018). A further drawback of using DNA-based metagenomic data to infer the biological functions potentially exploited by microbial populations is that the detected DNA may belong to dead organisms. However, an approach based on RNA sequencing would give a direct report of the functions achievable by the viable microbial populations. In grapevine, the study of the active fungal communities of internal grapevine wood by HTAS in extracted total RNA has been recently accomplished by Eichmeier et al. (2018).

The quantitative significance of next-generation sequencing data for microorganisms is often debated (Amend et al., 2010). Fortunately, we were able to compare the relative abundance of reads with the relative abundance of DNA of black-foot disease pathogens, and we observed significant positive correlation. From the fungal soilborne pathogens affecting grapevine, *Cylindrocarpon*-like asexual morphs associated with black-foot disease are among the most important limiting factor of the production worldwide (Halleen et al., 2006; Agustí-Brisach and Armengol, 2013). Therefore, *Cylindrocarpon*-like asexual morphs can be considered model pathogens to monitor the healthy status of the grapevine planting material when analyzing the fungal microbial composition of soil/rhizosphere samples.

Grapevine rootstocks have different susceptibilities toward pathogens, including trunk disease pathogens (Eskalen et al., 2001; Alaniz et al., 2010; Gramaje et al., 2010; Brown et al., 2013; Billones-Baaijens et al., 2014), which may be an important factor in shaping not only pathogens abundance but also entire communities. Nevertheless, we did not observe a clear correlation between known disease resistances in individual genotypes and the fungal communities, although *Cylindrocarpon*-like asexual morphs were found in lower abundance in 161-49 C rootstock by both high-throughput amplicon sequencing and qPCR approaches. The use of 161-49 C rootstock was previously recommended within an integrated management program for other grapevine trunk diseases, such as Petri disease and esca (Gramaje et al., 2010).

## Conclusion

We have studied the effects of genotype, year, sampling date, and location on bacterial and fungal communities in the grapevine rhizosphere. We found that grapevine genotype was the most important factor in shaping the microbiome in the mature vineyard. Many bacterial and fungal species were found in all rootstocks and in both locations in our study, demonstrating the existence of a “core” grape phylogeny that is independent of the growing region. Interestingly, the rhizosphere compartments of 140 Ru and 161-49 C rootstocks, the latter showing high tolerance to esca and Petri disease pathogens in previous research (Gramaje et al., 2010), harbored lower number of black-foot pathogens than the other grapevine rootstocks. Also of interest was the presence of high relative abundance of the genus *Bacillus* in both grapevine rootstocks, a bacterial genus recognized as biocontrol agents. A more comprehensive study is needed to decipher the cause of the rootstock microbiome selection and the mechanisms by which grapevines are able to shape their associated microbial community. Understanding the vast diversity of bacteria and fungi in the rhizosphere and the interactions between microbiota and grapevine will facilitate the development of future strategies for grapevine protection.

## Author Contributions

CB, MB, and DG conceived the study. All authors contributed to the data collection. CB, MB, GE, and DG performed the data interpretation and manuscript preparation. CB, MB, DG, GE, and ML performed the experiments. CB, MB, GE, and DG contributed to the bioinformatics data analysis. All authors critically reviewed and edited the manuscript, and approved its publication.

## Conflict of Interest Statement

The authors declare that the research was conducted in the absence of any commercial or financial relationships that could be construed as a potential conflict of interest.

## Footnotes

1 www.illumina.com

## Abbreviations

ITS: Internal transcribed spacer
OTU: Operational taxonomic unit
PCoA: Principal coordinate analysis
PCR: Polymerase chain reaction
PERMANOVA: Permutational multivariate analysis of variance
QIIME: Quantitative insights into microbial ecology
qPCR: Quantitative polymerase chain reaction
rRNA: Ribosomal RNA
SIMPER: Similarity percentages
