# Supplementary material for: The Fungal and Bacterial Rhizosphere Microbiome Associated With Grapevine Rootstock Genotypes in Mature and Young Vineyards
Source: Front Microbiol. 2019 May 22;10:1142. doi: 10.3389/fmicb.2019.01142 (PMC6538693; doi:10.3389/fmicb.2019.01142)

Supplementary Material

The fungal and bacterial rhizosphere microbiome associated with grapevine rootstock genotypes in mature and young vineyards

**Carmen Berlanas, Mónica Berbegal, Georgina Elena, Meriem Laidani, José Félix Cibriain, Ana Sagües-Sarasa, David Gramaje^*^**

*** Correspondence:** Corresponding Author: [david.gramaje@icvv.es](mailto:david.gramaje@icvv.es)

# Supplementary Tables

Supplementary Table 1. Information about rootstock selected in this study. The information is based in published studies (Hidalgo, 2002; Keller, 2010; Martínez-Cutillas et al., 1990).

| **Rootstock Type** | **Rootstock germplasm** | **Phylloxera resistance*** | **Nematode resistance** | **Grafted**  **Scion**  **Vigor** | **Tolerance** | | | **Ease of rooting** |
| --- | --- | --- | --- | --- | --- | --- | --- | --- |
|  |  |  |  |  | **Drought** | **Wet soil** | **Salt** |  |
| 110 R | *V. berlandieri* x *V. rupestris* | High | Poor | Medium | High | Low | Poor | Low |
| 140 Ru | *V. berlandieri* x *V. rupestris* | High | Low | High | High | Low | High | Poor |
| 1103 P | *V. berlandieri* x *V. rupestris* | High | Medium | Medium | Medium | Low | High | Medium |
| 41 B | *V. vinifera* x *V. berlandieri* | High | Poor | n.d. | High | Poor | Low | Medium |
| 161-49 C | *V. berlandieri* x *V. riparia* | Excellent. | Poor | Medium | Low | Low | Low | Low |

* Excellent > High > Medium > Poor > Low

**Supplementary Table 2.** Physicochemical properties, soil management practices and climate of the two vineyard soils examined in this study. Values represent the mean±SE.

|  | Aldea vineyard | Olite vineyard |
| --- | --- | --- |
| Coordinates | 42,234961º, -1,899365º | 42,252659º, 1,394441º |
| Altitude (m) | 347 | 396 |
| **Physicochemical properties** |  |  |
| pH | 8.4^a^±0.02 | 8.1 |
| P mg/100g* | 3.47±0.27 | 1.8±0.11 |
| K mg/100g | 15.52±0.59 | 17.3±0.44 |
| S mg/100g* | 4.37±0.38 | 0.9±0.27 |
| Mg mg/100g* | 25.57±0.29 | 15.0±0.26 |
| Mn mg/100g | 9.31±0.87 | 9.23±0.11 |
| Fe mg/100g* | 7.5±0.44 | 3.27±0.07 |
| Ca mg/100g* | 1570.92±220.81 | 1862.15±12.65 |
| Na mg/100g* | 6.08±0.19 | 1.46±0.05 |
| SOM%* | 0.74±0.02 | 1.75±0.01 |
| Clay% | 21.6±0.25 | 29.42±0.28 |
| Sand%* | 37.25±0.29 | 21.62±0.66 |
| Silt% | 41.12±0.05 | 49.0±0.41 |
| CO_3_Ca | 15.05±0.03 | 18.67±0.23 |
| CEC mekv/100g* | 9.7±0.23 | 21.25±0.10 |
| EC mS/cm | 0.15 | 0.16 |
| Assim. Ca mekv/100g* | 10.2±0.19 | 20.4±0.15 |
| Assim. Mg mekv/100g* | 1.47±0.03 | 0.8±0.04 |
| Soil temperature (ºC) (June) | 25.7 | 22 |
| Soil temperature (ºC) (November) | 10.5 | 10.6 |
| **Soil management practices** |  |  |
| Irrigation system | Drip irrigation | Drip irrigation |
| Fertilization | 1 application per year | 6 applications per year |
| Pest management practices | 5 spray treatments against powdery and downy mildew per year | 6 spray treatments against powdery and downy mildew per year |
| Herbicide treatment | Yes | No |
| **Climate** |  |  |
| Precipitation (mm) | 529 | 462 |
| Mean temperature (ºC) | 13.5 | 12.8 |

^a^Average of 4 replicates. Asterisk indicates statistically significant results (*P* < 0.05)

**Supplementary Table 3**. Number of reads, total OTUs, richness (Chao1 estimates of species richness) or diversity (Shannon’s index of diversity) indices expressed as average and standard deviation in the rootstock studied, for both bacteria and fungi analysis.

| **Aldea vineyard** | | | | | |
| --- | --- | --- | --- | --- | --- |
| Index | Bacteria | | | | |
|  | 110 R | 140 Ru | 1103 P | 41 B^*^ | 161 49 C |
| Reads | 29769.8±13217.1 | 36838.8±20139.9 | 30159.1±10864.9 | 32881.7±11279.7 | 28512.2±10966.7 |
| OTUs | 611 | 577 | 658 | 683 | 650 |
| Chao1 | 370.6±163.5 | 332.7±185.2 | 385.2±171.4 | 373.7±158.8 | 339.5±188.3 |
| Shannon | 4.0±0.7 | 4.1±0.5 | 4.2±0.2 | 4.2±0.3 | 4.3±0.2 |
|  | | | | | |
| Index | Fungi | | | | |
|  | 110 R | 140 Ru | 1103 P | 41 B | 161 49 C |
| Reads | 51139.7±29265.5 | 45952.4±28535.3 | 51436.2±28284.0 | 49659.7±16547.8 | 45420.4±29794.6 |
| OTUs | 246 | 259 | 250 | 241 | 224 |
| Chao1 | 168.9±31.2 | 183.4±43.1 | 176.5±31.9 | 180.3±52.0 | 176.2±47.0 |
| Shannon | 3.4±0.1 | 3.3±0.1 | 3.4±0.2 | 3.3±0.3 | 3.1±0.4 |

Richness and diversity indices calculated at an even sequencing depth of 1,000 sequences/sample for both bacteria and fungi

*Sample DG126 was removed from the analysis due to the low number of sequence reads

| **Olite vineyard** | | | | | |
| --- | --- | --- | --- | --- | --- |
| Index | Bacteria | | | | |
|  | 110 R* | 140 Ru | 1103 P | 41 B | 161 49 C |
| Reads | 37315.3±7632.7 | 4117.25±15790.9 | 39529±16251.5 | 34539.9±14862.6 | 58237.9±16430.6 |
| OTUs | 643 | 669 | 588 | 645 | 717 |
| Chao1 | 400.5±66.0 | 413.5±103.8 | 427.6±102.9 | 373.6±123.9 | 400.5±99.8 |
| Shannon | 4.2±0.4 | 4.3±0.2 | 4.3±0.07 | 4.1±0.3 | 4.2±0.2 |
|  | | | | | |
| Index | Fungi | | | | |
|  | 110 R | 140 Ru | 1103 P | 41 B | 161 49 C |
| Reads | 53623.8±23695.8 | 57160.5±27945.5 | 49059.2±21623.0 | 40862.6±23264.0 | 61027.7±33281.9 |
| OTUs | 311 | 359 | 355 | 333 | 372 |
| Chao1 | 189.2±64.2 | 223.7±33.6 | 213.7±51.3 | 202.1±33.1 | 224.4±43.9 |
| Shannon | 2.6±0.9 | 2.9±0.4 | 2.9±0.9 | 2.5±0.5 | 2.9±0.4 |

Richness and diversity indices calculated at an even sequencing depth of 1,000 sequences/sample for both bacteria and fungi

*Samples DG47 and DG48 were removed from the analysis due to the low number of sequence reads

**Supplementary Table 4.** Estimates of sample coverage and diversity indices at the genus level for bacteria and fungal profiles.

BACTERIA

| Sample ID | Good’s coverage | Chao1 richness | Shannon diversity |
| --- | --- | --- | --- |
| DG01 | 1.000 | 275.8 | 3.85 |
| DG02 | 0.995 | 265.9 | 3.73 |
| DG03 | 0.991 | 315.4 | 3.75 |
| DG04 | 0.999 | 220.3 | 3.70 |
| DG05 | 0.994 | 175.0 | 4.33 |
| DG06 | 0.988 | 155.1 | 4.21 |
| DG07 | 1.000 | 180.2 | 4.03 |
| DG08 | 0.981 | 210.1 | 3.85 |
| DG09 | 0.933 | 161.1 | 4.04 |
| DG10 | 0.921 | 250.4 | 4.10 |
| DG11 | 0.995 | 321.7 | 4.25 |
| DG12 | 0.973 | 335.0 | 4.11 |
| DG13 | 0.933 | 245.0 | 3.99 |
| DG14 | 0.933 | 245.1 | 3.88 |
| DG15 | 0.999 | 219.9 | 3.95 |
| DG16 | 0.987 | 240.9 | 3.80 |
| DG17 | 0.931 | 276.8 | 4.68 |
| DG18 | 0.910 | 299.9 | 3.75 |
| DG19 | 0.923 | 310.0 | 3.85 |
| DG20 | 0.962 | 325.4 | 4.15 |
| DG21 | 0.987 | 260.5 | 4.30 |
| DG22 | 0.987 | 305.4 | 4.25 |
| DG23 | 0.892 | 216.6 | 4.23 |
| DG24 | 0.900 | 351.8 | 4.11 |
| DG25 | 0.903 | 391.8 | 4.04 |
| DG26 | 0.987 | 255.0 | 3.95 |
| DG27 | 0.911 | 200.8 | 3.89 |
| DG28 | 0.987 | 428.9 | 3.99 |
| DG29 | 0.927 | 335.6 | 4.01 |
| DG30 | 0.987 | 350.4 | 3.97 |
| DG31 | 0.994 | 402.5 | 3.97 |
| DG32 | 0.991 | 265.7 | 3.99 |
| DG33 | 0.991 | 270.2 | 4.23 |
| DG34 | 1.000 | 221.4 | 4.26 |
| DG35 | 1.000 | 170.6 | 4.29 |
| DG36 | 0.995 | 170.7 | 3.95 |
| DG37 | 0.981 | 145.7 | 4.33 |
| DG38 | 0.988 | 143.6 | 3.98 |
| DG39 | 0.981 | 158.8 | 3.91 |
| DG40 | 0.991 | 230.4 | 3.80 |
| DG41 | 0.909 | 399.1 | 4.08 |
| DG42 | 1.000 | 225.7 | 4.09 |
| DG43 | 1.000 | 403.6 | 4.09 |
| DG44 | 0.909 | 175.9 | 4.12 |
| DG45 | 0.999 | 224.5 | 3.58 |
| DG46 | 0.987 | 214.4 | 3.42 |
| DG49 | 0.994 | 221.1 | 3.65 |
| DG50 | 0.895 | 215.9 | 3.66 |
| DG51 | 0.958 | 380.9 | 3.77 |
| DG52 | 0.910 | 235.9 | 4.10 |
| DG53 | 0.895 | 270.6 | 3.41 |
| DG54 | 0.899 | 390.7 | 3.64 |
| DG55 | 0.895 | 399.8 | 3.76 |
| DG56 | 0.920 | 448.1 | 3.80 |
| DG57 | 0.962 | 461.6 | 2.49 |
| DG58 | 0.958 | 365.7 | 3.67 |
| DG59 | 0.900 | 375.7 | 4.35 |
| DG60 | 0.910 | 378.6 | 4.15 |
| DG61 | 0.999 | 410.1 | 4.33 |
| DG62 | 0.855 | 419.3 | 4.21 |
| DG63 | 0.995 | 448.4 | 4.15 |
| DG66 | 0.981 | 465.6 | 4.21 |
| DG67 | 0.988 | 465.2 | 4.09 |
| DG68 | 0.981 | 410.4 | 4.13 |
| DG71 | 0.995 | 503.0 | 3.96 |
| DG72 | 0.995 | 499.8 | 3.95 |
| DG73 | 0.960 | 470.4 | 4.01 |
| DG76 | 0.995 | 518.5 | 4.10 |
| DG77 | 0.855 | 475.6 | 3.94 |
| DG78 | 1.000 | 460.7 | 4.12 |
| DG81 | 0.960 | 475.6 | 4.21 |
| DG82 | 0.994 | 500.1 | 4.15 |
| DG83 | 1.000 | 501.2 | 4.17 |
| DG86 | 0.884 | 426.6 | 4.68 |
| DG87 | 0.910 | 446.7 | 3.76 |
| DG88 | 0.899 | 455.8 | 3.85 |
| DG91 | 0.910 | 478.0 | 3.86 |
| DG92 | 0.899 | 480.9 | 4.02 |
| DG93 | 0.884 | 449.9 | 4.05 |
| DG96 | 0.905 | 515.6 | 4.25 |
| DG97 | 0.967 | 535.9 | 4.30 |
| DG98 | 0.899 | 462.1 | 4.29 |
| DG101 | 0.905 | 501.0 | 4.14 |
| DG102 | 0.884 | 508.0 | 4.21 |
| DG103 | 0.905 | 445.5 | 4.20 |
| DG106 | 0.904 | 456.8 | 4.19 |
| DG107 | 0.884 | 425.9 | 4.15 |
| DG108 | 0.899 | 485.9 | 4.23 |
| DG111 | 0.994 | 470.2 | 3.54 |
| DG112 | 0.981 | 465.7 | 4.24 |
| DG113 | 0.889 | 480.8 | 4.51 |
| DG116 | 0.995 | 501.8 | 4.52 |
| DG117 | 0.949 | 525.0 | 4.35 |
| DG118 | 1.000 | 485.1 | 4.35 |
| DG121 | 0.949 | 519.4 | 4.12 |
| DG122 | 0.973 | 490.5 | 4.27 |
| DG123 | 0.973 | 485.7 | 4.28 |
| DG127 | 0.995 | 405.2 | 4.35 |
| DG128 | 0.991 | 406.3 | 3.98 |
| DG131 | 0.889 | 410.0 | 4.11 |
| DG132 | 0.889 | 445.0 | 4.13 |
| DG133 | 0.995 | 443.7 | 4.01 |
| DG136 | 0.911 | 448.9 | 4.11 |
| DG137 | 0.857 | 540.1 | 4.24 |
| DG138 | 0.908 | 525.3 | 4.31 |
| DG141 | 0.912 | 250.2 | 4.15 |
| DG142 | 0.900 | 251.2 | 4.13 |
| DG143 | 0.900 | 445.6 | 4.11 |
| DG146 | 0.851 | 510.5 | 4.10 |
| DG147 | 0.896 | 509.8 | 4.21 |
| DG148 | 0.884 | 505.0 | 3.76 |
| DG151 | 0.911 | 475.0 | 4.21 |
| DG152 | 0.904 | 549.5 | 4.20 |
| DG153 | 0.893 | 234.4 | 4.11 |
| DG156 | 0.884 | 421.3 | 4.19 |
| DG157 | 0.896 | 446.7 | 4.19 |
| DG158 | 0.911 | 447.5 | 4.13 |
| Average | 0.945 | 370.0 | 4.00 |

FUNGI

| Sample ID | Good’s coverage | Chao1 richness | Shannon diversity |
| --- | --- | --- | --- |
| DG01 | 0.966 | 103.0 | 3.41 |
| DG02 | 0.991 | 130.5 | 3.43 |
| DG03 | 0.991 | 130.6 | 3.50 |
| DG04 | 0.872 | 155.7 | 3.52 |
| DG05 | 0.952 | 156.0 | 3.30 |
| DG06 | 0.930 | 175.1 | 3.54 |
| DG07 | 0.891 | 140.1 | 3.57 |
| DG08 | 0.988 | 138.3 | 3.55 |
| DG09 | 0.875 | 139.0 | 3.55 |
| DG10 | 0.991 | 170.3 | 3.83 |
| DG11 | 0.873 | 140.5 | 3.33 |
| DG12 | 0.872 | 110.4 | 3.34 |
| DG13 | 0.829 | 119.4 | 3.51 |
| DG14 | 0.973 | 172.0 | 3.50 |
| DG15 | 0.853 | 175.1 | 3.54 |
| DG16 | 0.875 | 165.2 | 2.55 |
| DG17 | 0.855 | 180.3 | 1.80 |
| DG18 | 0.971 | 196.2 | 2.68 |
| DG19 | 0.891 | 163.7 | 2.76 |
| DG20 | 0.992 | 90.8 | 1.80 |
| DG21 | 0.829 | 241.0 | 3.60 |
| DG22 | 0.856 | 185.6 | 3.25 |
| DG23 | 0.905 | 190.7 | 3.33 |
| DG24 | 0.875 | 200.3 | 3.34 |
| DG25 | 0.971 | 204.1 | 3.50 |
| DG26 | 0.849 | 170.1 | 3.41 |
| DG27 | 0.849 | 175.8 | 3.34 |
| DG28 | 0.853 | 175.6 | 3.32 |
| DG29 | 0.875 | 147.5 | 3.30 |
| DG30 | 0.905 | 223.6 | 3.29 |
| DG31 | 0.904 | 215.8 | 3.04 |
| DG32 | 0.890 | 216.0 | 3.10 |
| DG33 | 0.930 | 165.3 | 3.12 |
| DG34 | 0.991 | 112.4 | 3.43 |
| DG35 | 0.973 | 128.5 | 3.11 |
| DG36 | 0.944 | 141.1 | 3.13 |
| DG37 | 0.930 | 225.5 | 3.18 |
| DG38 | 0.952 | 165.5 | 3.15 |
| DG39 | 0.872 | 225.8 | 3.04 |
| DG40 | 0.855 | 148.0 | 3.02 |
| DG41 | 0.930 | 173.0 | 3.14 |
| DG42 | 0.973 | 164.3 | 3.21 |
| DG43 | 0.905 | 160.2 | 3.22 |
| DG44 | 0.966 | 135.1 | 3.24 |
| DG45 | 0.872 | 138.5 | 3.14 |
| DG46 | 0.837 | 90.5 | 2.75 |
| DG47 | 0.973 | 155.8 | 2.87 |
| DG48 | 0.857 | 161.0 | 3.10 |
| DG49 | 0.952 | 162.5 | 3.05 |
| DG50 | 0.857 | 165.6 | 3.03 |
| DG51 | 0.835 | 225.5 | 3.09 |
| DG52 | 0.849 | 205.4 | 2.87 |
| DG53 | 0.880 | 185.9 | 2.91 |
| DG54 | 0.991 | 224.0 | 3.36 |
| DG55 | 0.890 | 203.6 | 3.34 |
| DG56 | 0.830 | 140.7 | 2.99 |
| DG57 | 0.829 | 148.6 | 3.13 |
| DG58 | 0.900 | 181.5 | 3.12 |
| DG59 | 0.831 | 182.6 | 3.50 |
| DG60 | 0.904 | 159.1 | 3.84 |
| DG61 | 0.836 | 208.3 | 3.34 |
| DG62 | 0.844 | 206.4 | 3.59 |
| DG63 | 0.849 | 223.5 | 3.56 |
| DG66 | 0.904 | 244.9 | 3.54 |
| DG67 | 0.896 | 189.0 | 3.51 |
| DG68 | 0.966 | 190.7 | 3.44 |
| DG71 | 0.896 | 250.7 | 3.44 |
| DG72 | 0.857 | 211.8 | 3.82 |
| DG73 | 0.880 | 188.7 | 3.58 |
| DG76 | 0.844 | 177.6 | 3.59 |
| DG77 | 0.893 | 207.3 | 3.33 |
| DG78 | 0.971 | 185.4 | 3.44 |
| DG81 | 0.833 | 250.5 | 3.58 |
| DG82 | 0.930 | 220.0 | 2.65 |
| DG83 | 0.845 | 205.0 | 2.51 |
| DG86 | 0.952 | 225.7 | 2.65 |
| DG87 | 0.911 | 210.1 | 2.23 |
| DG88 | 0.893 | 208.4 | 2.51 |
| DG91 | 0.860 | 203.4 | 3.65 |
| DG92 | 0.860 | 192.1 | 3.62 |
| DG93 | 0.930 | 191.9 | 3.10 |
| DG96 | 0.829 | 235.0 | 3.12 |
| DG97 | 0.893 | 240.4 | 3.70 |
| DG98 | 0.911 | 202.5 | 2.90 |
| DG101 | 0.899 | 191.5 | 3.55 |
| DG102 | 0.896 | 226.0 | 3.43 |
| DG103 | 0.880 | 212.2 | 3.13 |
| DG106 | 0.902 | 203.4 | 3.15 |
| DG107 | 0.956 | 221.3 | 3.12 |
| DG108 | 0.938 | 175.1 | 3.26 |
| DG111 | 0.944 | 183.6 | 3.31 |
| DG112 | 0.934 | 240.9 | 2.92 |
| DG113 | 0.845 | 215.3 | 3.34 |
| DG116 | 0.893 | 186.6 | 3.33 |
| DG117 | 0.881 | 210.0 | 2.93 |
| DG118 | 0.872 | 245.5 | 3.42 |
| DG121 | 0.833 | 220.4 | 3.32 |
| DG122 | 0.911 | 231.5 | 3.24 |
| DG123 | 0.905 | 227.6 | 3.25 |
| DG126 | 0.846 | 214.2 | 3.21 |
| DG127 | 0.890 | 223.3 | 3.24 |
| DG128 | 0.841 | 201.1 | 3.26 |
| DG131 | 0.841 | 223.1 | 2.24 |
| DG132 | 0.842 | 220.6 | 3.35 |
| DG133 | 0.833 | 218.7 | 3.41 |
| DG136 | 0.896 | 180.6 | 3.45 |
| DG137 | 0.966 | 220.5 | 3.23 |
| DG138 | 0.967 | 221.9 | 3.24 |
| DG141 | 0.923 | 254.9 | 3.25 |
| DG142 | 0.994 | 230.7 | 3.25 |
| DG143 | 0.900 | 215.0 | 3.03 |
| DG146 | 0.847 | 240.1 | 2.99 |
| DG147 | 0.848 | 222.0 | 3.11 |
| DG148 | 0.988 | 191.2 | 3.23 |
| DG151 | 0.841 | 223.3 | 3.14 |
| DG152 | 0.993 | 248.9 | 3.15 |
| DG153 | 0.960 | 190.3 | 3.25 |
| DG156 | 0.950 | 222.8 | 3.23 |
| DG157 | 0.860 | 234.8 | 3.11 |
| DG158 | 0.983 | 241.9 | 3.24 |
| Average | 0.901 | 190.6 | 3.21 |

**Supplementary Table 5.** Experimental factors predicting α- and β-diversity of rhizosphere associated bacterial and fungal communities between vineyards

ANOVA, analysis of variance

All *P* values were corrected for multiple comparisons using the sequential Bonferroni correction. Significance was assessed using Type III ANOVA. Bold values indicate statistically significant results after correction for multiple comparisons, *P* < 0.05.

|  | α-diversity | |  | β-diversity |
| --- | --- | --- | --- | --- |
|  | Shannon | Chao1 |  | Bray Curtis |
| Bacteria | *F_1,115_* = 0.27  *P* = 0.7840 | *F_1,115_* = 1.42  *P* = 0.1567 |  | *R*^2^ = 0.19  *P* = 0.1134 |
| Fungi | *F_1,117_* = 2.15 | *F_1,117_* = 1.37 |  | *R^2^* = 0.69 |
|  | ***P* = 0.033** | *P* = 0.1724 |  | ***P* < 0.001** |

**Supplementary Table 6.** Similarity percentages (SIMPER) analysis determines the bacterial phyla **(A)** and genera **(B)**, and fungal phyla **(C)** and genera **(D)** contributions to the dissimilarity among rootstocks in the rhizosphere. In the upper part of the table the rootstock pairwise comparison of average dissimilarity percentage has been reported. In the lower part, the overall top one, two or three phyla/genera contributing to the pairwise dissimilarity were listed, reporting in parenthesis their relative contribution to the observed dissimilarity expressed as percentage.

**(A)** SIMPER analysis determined the bacterial phyla contributions in Aldea and Olite vineyards

| **ALDEA** | **110 R** | **140 Ru** | **1103 P** | **41 B** | **161 49 C** |
| --- | --- | --- | --- | --- | --- |
| **110 R** |  | 9.2 | 16.1^#^ | 7.2 | 5.5^†^ |
| **140 Ru** | Latescibacteria (21.3)  Firmicutes (19.3)  Planctomycetes (11.2) |  | 8.2 | 10.1^#^ | 10.7^#^ |
| **1103 P** | Acidobacteria (24.5)  Firmicutes (15.6)  Gemmatimonadetes (11.3) | Acidobacteria (31.5)  Firmicutes (19.5)  Planctomycetes (9.3) |  | 11.0^#^ | 18.1^#^ |
| **41 B** | Firmicutes (23.1)  Nitrospirae (21.6)  Actinobacteria (8.5) | Firmicutes (24.4)  Acidobacteria (15.6)  Latescibacteria (11.1) | Actinobacteria (19.4)  Bacteroidetes (13.4)  Planctomycetes (12.2) |  | 8.4 |
| **161 49 C** | Firmicutes (29.5)  Acidobacteria (21.5)  Nitrospirae (13.8) | Acidobacteria (32.4)  Firmicutes (18.6)  candidatedivisionWPS_1 (13.4) | Firmicutes (30.6)  candidatedivisionWPS_1 (25.6)  Verrucomicrobia (16.7) | Firmicutes (29.5)  Actinobacteria (17.8)  Acidobacteria (8.9) |  |
|  |  |  |  |  |  |
| **OLITE** | **110 R** | **140 Ru** | **1103 P** | **41 B** | **161 49 C** |
| **110 R** |  | 8.1 | 9.5 | 7.6 | 10.3^#^ |
| **140 Ru** | No significant phyla |  | 3.7 | 2.5 | 5.0 |
| **1103 P** | Nitrospirae (14.4) | Nitrospirae (22.3)  Acidobacteria (17.8) |  | 5.1 | 1.8^†^ |
| **41 B** | Parcubacteria (16.5)  Acidobacteria (13.2) | Acidobacteria (25.0) | Acidobacteria (27.6) |  | 4.8 |
| **161 49 C** | Acidobacteria (21.6) | Acidobacteria (24.3)  Nitrospirae (14.4)  Ud_Bacteria (11.0) | Acidobacteria (25.4)  Chlamydiae (10.1) | No significant phyla |  |

^#^ Rootstock-pairs showing dissimilarity in phyla distribution higher than 10%

^†^ Rootstock-pair showing the lowest dissimilarity observed in phyla distribution

**(B)** SIMPER analysis determined the bacterial genera contributions in Aldea and Olite vineyards

| **ALDEA** | **110 R** | **140 Ru** | **1103 P** | **41 B** | **161 49 C** |
| --- | --- | --- | --- | --- | --- |
| **110 R** |  | 5.5 | 14.8^#^ | 9.7 | 12.8^#^ |
| **140 Ru** | *Corynebacterium* (24.3)  Ud_Microbacteriaceae (23.5)  Nocardioides (11.7) |  | 9.9 | 6.3 | 6.1 |
| **1103 P** | *Gp6* (26.0)  Ud_Betaproteobacteria (25.6)  *Gemmatimonas* (21.1) | *Bacillus* (26.6)  *Gp4* (23.4)  *Gp6* (14.2) |  | 4.9 | 4.7^†^ |
| **41 B** | *Ilumatobacter* (23.6)  *Propionibacterium* (22.5)  *Rubrobacter* (21.3) | *Bacillus* (25.7)  *Nocardioides* (14.5)  Ud_Bacillales (11.0) | *Mycobacterium* (25.1)  Ud_Betaproteobacteria (22.0)  *Ilumatobacter* (9.8) |  | 5.1 |
| **161 49 C** | Ud_Bacillales (25.6)  *Bacillus* (12.3)  Ud_Rhodocyclaceae (12.1) | *Bacillus* (24.5)  *Gaiella* (15.5)  *GP4* (8.8) | *Bacillus* (27.8)  *Serratia* (18.4)  *Pesudomonas* (14.0) | *Bacillus* (29.8)  *Ilumatobacter* (22.6)  *Propionibacterium* (20.5) |  |
|  |  |  |  |  |  |
| **OLITE** | **110 R** | **140 Ru** | **1103 P** | **41 B** | **161 49 C** |
| **110 R** |  | 3.6 | 3.8 | 2.5 | 2.1^†^ |
| **140 Ru** | *Aquicella* (15.4)  *Flavobacterium* (9.8)  Ud_Bradyrhizobiaceae (9.7) |  | 3.0 | 4.1 | 3.1 |
| **1103 P** | *Aridibacter* (21.0)  *Aquicella* (17.6)  *Chitinophaga* (15.0) | *Aridibacter* (17.7)  Ud_Cytophagales (12.3)  *Vasilyevaea* (6.7) |  | 6.8 | 3.5 |
| **41 B** | No significant genera | *Gp6* (14.4)  *Povalibacter* (14.1)  Ud_Proteobacteria (10.9) | *Gp10* (17.0)  *Gemmata* (11.4)  *Flavobacterium* (11.2) |  | 6.7 |
| **161 49 C** | *Aridibacter* (16.7)  Ud_Acidobacteria (16.1)  *Gp5* (11.4) | *Gp6* (15.4)  *Aridibacter* (15.2)  *Blastocatella* (13.4) | *Gp10* (19.0)  *Sphingomonas* (15.9)  *Mycobacterium* (15.6) | Ud_Sphingomonadales (23.4)  *Aeromicrobium* (21.0)  *Povalibacter* (14.5) |  |

^#^ Rootstock-pairs showing dissimilarity in genera distribution higher than 10%

^†^ Rootstock-pair showing the lowest dissimilarity observed in genera distribution

**(C)** SIMPER analysis determined the fungal phyla contributions in Aldea and Olite vineyards

| **ALDEA** | **110 R** | **140 Ru** | **1103 P** | **41 B** | **161 49 C** |
| --- | --- | --- | --- | --- | --- |
| **110 R** |  | 8.2 | 16.2^#^ | 6.6^†^ | 25.3^#^ |
| **140 Ru** | Ascomycota (29.3)  Basidiomycota (24.1)  Zygomycota (17.8) |  | 9.8 | 22.7^#^ | 21.0^#^ |
| **1103 P** | Ascomycota (6.3) | Basidiomycota (30.8)  Zygomycota (9.2) |  | 25.9^#^ | 12.8^#^ |
| **41 B** | Basiodiomycota (22.9)  Ascomycota (12.8)  Glomeromycota (7.0) | Basidiomycota (22.4)  Zygomycota (16.8)  Ascomycota (8.4) | Glomeromycota (19.4)  Basiodiomycota (12.4)  Ascomycota (6.1) |  | 29.7^#^ |
| **161 49 C** | Ascomycota (27.5)  Zygomycota (13.5) | Basidiomycota (35.5) | Glomeromycota (22.1) | Glomeromycota (19.5)  Zygomycota (13.5)  Ascomycota (13.5) |  |
|  |  |  |  |  |  |
| **OLITE** | **110 R** | **140 Ru** | **1103 P** | **41 B** | **161 49 C** |
| **110 R** |  | 2.2 | 1.9 | 3.2 | 1.5^†^ |
| **140 Ru** | Basidiomycota (8.1) |  | 1.7 | 2.9 | 3.3 |
| **1103 P** | No significant phyla | No significant phyla |  | 5.6 | 8.0 |
| **41 B** | No significant phyla | No significant phyla | No significant phyla |  | 9.2 |
| **161 49 C** | Basidiomycota (8.8) | No significant phyla | No significant phyla | Basidiomycota (5.5) |  |

^#^ Rootstock-pairs showing dissimilarity in phyla distribution higher than 10%

^†^ Rootstock-pair showing the lowest dissimilarity observed in phyla distribution

**(D)** SIMPER analysis determined the fungal genera contributions in Aldea and Olite vineyards

| **ALDEA** | **110 R** | **140 Ru** | **1103 P** | **41 B** | **161 49 C** |
| --- | --- | --- | --- | --- | --- |
| **110 R** |  | 7.2 | 7.1 | 7.8 | 12.7^#^ |
| **140 Ru** | Ud_Auriculariales (19.0)  *Geopyxis* (15.5)  *Psathyrella* (15.1) |  | 6.5 | 6.6 | 16.1^#^ |
| **1103 P** | Ud_ Pleosporales (17.5)  *Sporormiella* (15.9)  *Articulospora* (15.3) | *Clonostachys* (15.5)  *Lecanicillium* (14.6)  *Scutellinia* (10.3) |  | 6.3^†^ | 12.9^#^ |
| **41 B** | Ud_Giomeraceae (13.1)  *Gongronella* (13.0)  *Geopyxis* (13.0) | *Clonostachys* (14.4)  *Lecanicillium* (12.5)  *Psathyrella* (9.1) | *Clonostachys* (8.1)  Ud_Glomeraceae (8.1)  Ud_Nectriaceae (8.0) |  | 17.5^#^ |
| **161 49 C** | *Geopyxis* (16.5)  Ud_Glomeraceae (16.1)  *Psathyrella* (15.6) | *Clonostachys* (9.4)  *Cryptococcus* (9.1)  *Davidiella* (9.0) | *Clonostachys* (9.2)  *Cryptococcus* (9.2)  *Lecanicillium* (9.2) | *Cryptococcus* (8.8)  *Davidiella* (8.7)  *Lecanicillium* (8.7) |  |
|  |  |  |  |  |  |
| **OLITE** | **110 R** | **140 Ru** | **1103 P** | **41 B** | **161 49 C** |
| **110 R** |  | 4.1 | 8.2 | 8.5 | 8.7 |
| **140 Ru** | *Gymnopus* (5.6)  *Spizellomyces* (4.6)  *Pseudogymnoascus* (4.3) |  | 4.2 | 2.7^†^ | 7.1 |
| **1103 P** | *Calcarisporiella* (8.7) | *Geopyxis* (14.3) *Calcarisporiella* (11.7)  *Scytalidium* (10.5) |  | 12.8^#^ | 8.8 |
| **41 B** | *Clavaria* (11.2)  *Scytalidium* (6.7)  *Penicillium* (4.5) | *Geopyxis* (17.5) *Scytalidium* (14.0)  *Gymnopus* (13.8) | Ud_Basiodiomycota (6.1)  Ud_Ceratobasidiaceae (5.5)  *Calcarisporiella* (4.5) |  | 4.1 |
| **161 49 C** | Ud_Auriculariales (11.2)  *Spizellomyeces* (10.3)  *Geopyxis* (6.6) | *Scutellinia* (13.4)  Ud_Auriculariales (13.2)  *Gymnopus* (5.7) | *Scutellinia* (10.1)  Ud_Basidiomycota (7.6)  *Gymnoascus* (6.7) | Ud_Auriculariales (12.5)  *Gymnoascus* (7.6)  *Scytalidium* (5.9) |  |

^#^ Rootstock-pairs showing dissimilarity in genera distribution higher than 10%

^†^ Rootstock-pair showing the lowest dissimilarity observed in genera distribution

**Supplementary Table 7.** Bacterial OTUs that were unique in each of the sample type.

|  | Rootstock | | | | |
| --- | --- | --- | --- | --- | --- |
| Vineyard | 110 R | 140 Ru | 1103 P | 41 B | 161 49C |
| Aldea |  |  |  |  |  |
|  | Gp21 | Mobiluncus | Terriglobus | Pilimelia | Turicella |
|  | Pyrinomonas | Planosporangium | ud-Dermacoccaceae | Gordonia | ud-Demequinaceae |
|  | Phytomonospora | Plantactinospora | Pseudoclavibacter | Xylanibacterium | Barrientosiimonas |
|  | Crossiella | Actinokineospora | Enteractinococcus | Micropruina | Dietzia |
|  | Atopobium | Prevotella | Zhihengliuella | ud-Prolixibacteraceae | Amnibacterium |
|  | Vibrionimonas | Rubrivirga | Rugosimonospora | Capnocytophaga | Krasilnikovia |
|  | Thermosporothrix | ud-Alicyclobacillaceae | Tessaracoccus | Empedobacter | Brooklawnia |
|  | Elusimicrobium | Thermicanus | Thermocatellispora | Epilithonimonas | Thermobispora |
|  | Halobacillus | Abiotrophia | Bacteroides | Kyrpidia | Imperialibacter |
|  | Marininema | Acetatifactor | Paludibacter | Geomicrobium | Filimonas |
|  | Hespellia | ClostridiumXlVb | Tannerella | Piscibacillus | Falsibacillus |
|  | Peptoniphilus | ud-Lentisphaerae | Bhargavaea | Gemella | Guggenheimella |
|  | Faecalibacterium | Starkeya | Oxobacter | Jeotgalicoccus | Anaerovorax |
|  | Leptotrichia | Pandoraea | Eisenbergiella | Desemzia | Lachnoanaerobaculum |
|  | Albidovulum | Tepidiphilus | Pelotomaculum | Dolosigranulum | Stomatobaculum |
|  | Sandarakinorhabdus | Simonsiella | ClostridiumIV | Streptococcus | Halobacteroides |
|  | Burkholderia | ud-Desulfobacteraceae | Oscillibacter | Anaerobacter | ud-Halobacteroidaceae |
|  | Kingella | Alishewanella | Anoxybacter | Natronincola | Ignavibacterium |
|  | Rivicola | ud-Chromatiaceae | Megasphaera | Tepidanaerobacter | Pelagibacterium |
|  | Desulfohalobium | Vulcaniibacterium | Camelimonas | Dehalobacter | Hansschlegelia |
|  | ud-Desulfohalobiaceae | Leptonema | Prosthecomicrobium | ud-Peptococcaceae2 | Aquamicrobium |
|  | Corallococcus | Leptospira | Rhodomicrobium | Pseudobacteroides | Phreatobacter |
|  | Leclercia | Spirochaeta | Methyloligella | Dialister | Defluviimonas |
|  | Marinomonas | ud-Spirochaetaceae | Stella | Fusobacterium | Falsirhodobacter |
|  | ud-Oceanospirillales | Fervidobacterium | Nitrospirillum | Cereibacter | Rhodobacter |
|  | Alkanindiges |  | Anaplasma | Elioraea | Acidisoma |
|  | Moraxella |  | Advenella | Oceanibaculum | Defluviicoccus |
|  | Aspromonas |  | Malikia | Tistlia | Pigmentiphaga |
|  | Acholeplasma |  | Undibacterium | Limnobacter | Sulfurisoma |
|  | Subdivision5_genera_incertae_sedis | | ud-Desulfovibrionaceae | Thiobacter | Halobacteriovorax |
|  |  |  | ud-Desulfovibrionales | Chitinibacter | Desulfocapsa |
|  |  |  | Ignatzschineria | Snodgrassella | Desulfuromonas |
|  |  |  | Cloacibacillus | ud-Nitrosomonadaceae | ud-Syntrophaceae |
|  |  |  | ud-Synergistaceae | Azonexus | Campylobacter |
|  |  |  |  | Georgfuchsia | Raoultella |
|  |  |  |  | Sulfuricurvum | ud-Methylococcaceae |
|  |  |  |  | Sulfurimonas | Luteibacter |
|  |  |  |  | Aggregatibacter | Limisphaera |
|  |  |  |  | ud-Pasteurellaceae | |
|  |  |  |  | ud-Pseudomonadales |  |
| Olite |  |  |  |  |  |
|  | Actinospica | Flaviflexus | ud-Cryptosporangiaceae | Citricoccus | Pyrinomonas |
|  | Stackebrandtia | Frigoribacterium | Rubricoccus | Zhihengliuella | Pseudoclavibacter |
|  | ud-Glycomycetaceae | Enteractinococcus | ud-Paenibacillaceae2 | Capnocytophaga | Polymorphospora |
|  | Amnibacterium | Micropruina | ud-Aerococcaceae | Asinibacterium | Rugosimonospora |
|  | Plantibacter | Microbispora | Flavonifractor | Solitalea | Brooklawnia |
|  | ud-Bifidobacteriaceae | Atopobium | Pseudoflavonifractor | Thermicanus | Odoribacter |
|  | ud-Deinococcales | Alicyclobacillus | Vallitalea | Saccharibacillus | Algoriphagus |
|  | ud-Enterococcaceae | Aeribacillus | Undibacterium | Chungangia | Nibrella |
|  | Lactococcus | Geomicrobium | Psychrobacter | Alloiococcus | Imperialibacter |
|  | Anaerosolibacter | ud-Bacillales_incertae_sedis | Spirochaeta | Anaerobacter | ud-Flammeovirgaceae |
|  | Sporanaerobacter | Aerococcus | Subdivision5_genera_incertae_sedis | ud-Clostridiaceae2 | Filimonas |
|  | Garciella | Fonticella |  | Stomatobaculum | Heliimonas |
|  | Desulfitispora | Tepidanaerobacter |  | Peptoniphilus | Arcticibacter |
|  | ud-Peptococcaceae1 | Guggenheimella |  | Butyricicoccus | ud-Chloroflexaceae |
|  | Ralstonia | Acetatifactor |  | ud-Erysipelotrichaceae | ud-Deinococcaceae |
|  | Hydrogenophilus | Blautia |  | Ignavibacterium | Elusimicrobium |
|  | Arcobacter | Sporotomaculum |  | Oligosphaera | Candidatus Endomicrobium |
|  | Marinobacter | ud-Peptococcaceae2 | | ud-Hyphomonadaceae | Kyrpidia |
|  | Shewanella | Gemmiger |  | Pandoraea | Halobacillus |
|  | Kosakonia | Hydrogenoanaerobacterium | | Massilia | Marininema |
|  | Aggregatibacter | Oscillibacter |  | Hafnia | Abiotrophia |
|  | Leptonema | Syntrophomonas |  | Klebsiella | ud-Carnobacteriaceae |
|  |  | Gluconobacter |  | ud-Oceanospirillales | Oxobacter |
|  |  | Castellaniella |  | Xiphinematobacter | ud-Clostridiaceae3 |
|  |  | Sphaerotilus |  |  | Anaerovorax |
|  |  | Azoarcus |  |  | Anaerobacterium |
|  |  | Dechloromonas |  |  | Anoxybacter |
|  |  | Celerinatantimonas |  |  | Selenomonas |
|  |  | Methylobacter |  |  | Camelimonas |
|  |  | Aspromonas |  |  | Rhodomicrobium |
|  |  | SR1_genera_incertae_sedis | |  | Aquamicrobium |
|  |  |  |  |  | Alsobacter |
|  |  |  |  |  | Phreatobacter |
|  |  |  |  |  | Methyloligella |
|  |  |  |  |  | Falsirhodobacter |
|  |  |  |  |  | Rhodobacter |
|  |  |  |  |  | Acidisoma |
|  |  |  |  |  | Magnetospirillum |
|  |  |  |  |  | Orientia |
|  |  |  |  |  | Sandarakinorhabdus |
|  |  |  |  |  | Advenella |
|  |  |  |  |  | Inhella |
|  |  |  |  |  | Malikia |
|  |  |  |  |  | Roseateles |
|  |  |  |  |  | Paraherbaspirillum |
|  |  |  |  |  | Thiobacillus |
|  |  |  |  |  | ud-Neisseriaceae |
|  |  |  |  |  | Halobacteriovorax |
|  |  |  |  |  | Corallococcus |
|  |  |  |  |  | ud-Syntrophaceae |
|  |  |  |  |  | Campylobacter |
|  |  |  |  |  | ud-Methylococcaceae |
|  |  |  |  |  | Leptospira |
|  |  |  |  |  | Anaeroplasma |
|  |  |  |  |  | Limisphaera |

**Supplementary Table 8**. Fungal OTUs that were unique in each of the simple type.

|  | Rootstock | | | | |
| --- | --- | --- | --- | --- | --- |
| Vineyard | 110 R | 140 Ru | 1103 P | 41 B | 161 49 C |
| Aldea |  |  |  |  |  |
|  | Astraeus | Bipolaris | Amaurodon | Camarosporium | Ascosphaera |
|  | Debaryomyces | Canalisporium | Amphinema | Chaetomiaceae_ud | Capnodiales_ud |
|  | Glomerales_ud | Colletotrichum | Cadophora | Cyathus | Cladophialophora |
|  | Hymenoscyphus | Crepidotus | Geotrichum | Dothiorella | Devriesia |
|  | Incertae_sedis_12_ud | Cytospora | Incertae_sedis_26_ud | Haematonectria | Morchellaceae_ud |
|  | Lecythophora | Guehomyces | Inocybe | Hebeloma | Mycenastrum |
|  | Leotiomycetes_ud | Gymnopus | Phanerochaete | Lyophyllum | Teratosphaeriaceae_ud |
|  | Lycoperdaceae_ud | Hypocrea | Pholiota | Oidiodendron | Hyaloscyphaceae_ud |
|  | Neophaeosphaeria | Lacrymaria | Pilaira | Ophiosphaerella | Incertae_sedis_25 |
|  | Pyrenochaeta | Neofusicoccum | Pilidium | Phaeosphaeriaceae_ud | Filobasidiales |
|  | Rinodina | Pisolithus | Podospora | Scleroderma |  |
|  | Sarcinomyces | Polyporales_ud | Pyronemataceae_ud | Tomentella |  |
|  | Sphaeropsis | Pringsheimia | Sarocladium | Tricholomataceae_ud | |
|  | Teloschistaceae_ud | Thecaphora | Stagonospora | Typhula |  |
|  | Tulostoma | Helotiaceae_ud | Ascobolaceae | Mycosphaerellaceae_ud | |
|  | Teloschistaceae_ud | Strophariaceae | Diatrypaceae | Verrucariaceae_ud |  |
|  | Ascomycota_ud | Filobasidiaceae | Pleurotaceae | Pezizaceae |  |
|  | Volvariella | Incertae_sedis_12 | Ambisporaceae | Verrucaria |  |
|  |  | Pezizales | Cantharellales |  |  |
|  |  | Verticillium | Wallemia |  |  |
|  |  | Xenasmatella | Zygosaccharomyces | |  |
|  |  | Xylariaceae_ud |  |  |  |
| Olite |  |  |  |  |  |
|  | Auricularia | Alnicola | Arthroascus | Ambisporaceae_ud | Ascochyta |
|  | Corticiales_ud | Ampelomyces | Atheliaceae_ud | Amphinema | Byssomerulius |
|  | Dissoconium | Annulohypoxylon | Battarrea | Backusella | Cephalotheca |
|  | Eutypa | Athelia | Bulleromyces | Caloplaca | Cistella |
|  | Geopora | Calocybe | Ceratobasidium | Crepidotus | Claroideoglomus |
|  | Glarea | Cystolepiota | Clavicipitaceae_ud | Cristinia | Cochliobolus |
|  | Leotiomycetes_ud | Eupenicillium | Crocicreas | Eucasphaeria | Exobasidiomycetes_ud |
|  | Leveillula | Fibroporia | Didymosphaeria | Gliomastix | Gnomonia |
|  | Lycoperdaceae_ud | Gymnoascaceae_ud | Entyloma | Lachnella | Hypoxylon |
|  | Paurocotylis | Heterobasidion | Gloeophyllum | Mycena | Incertae_sedis_12_ud |
|  | Phaeocytostroma | Neostagonospora | Hemimycena | Neoerysiphe | Incertae_sedis_2_ud |
|  | Phialocephala | Plectania | Lachnum | Neofusicoccum | Leucopaxillus |
|  | Septoglomus | Pluteus | Lalaria | Parasola | Lichtheimia |
|  | Stagonospora | Polyscytalum | Millerozyma | Parasola | Lophodermium |
|  | Thanatephorus | Pringsheimia | Monacrosporium | Rosellinia | Monographella |
|  | Thelonectria | Pseudovalsaria | Pilaira | Simplicillium | Montagnulaceae_ud |
|  | Tulostoma | Pyrenochaetopsis | Psathyrellaceae_ud | Teloschistaceae_ud | Mycenastrum |
|  | Orbiliomycetes | Sphaerulina | Saccharomycetaceae_ud | Tylospora | Mycosphaerellaceae_ud |
|  |  | Tapinella | Sebacinales_ud | Microbotryomycetes | Myriodontium |
|  |  | Diatrypaceae | Sphaeropsis | Ustilaginales | Nemania |
|  |  | Marasmiaceae | Stropharia | Verticillium | Oudemansiella |
|  |  | Incertae_sedis_28 | Blumeria |  | Phlebia |
|  |  | Archaeorhizomycetes | Helotiaceae_ud |  | Plagiostoma |
|  |  | Cephalothecaceae | Incertae_sedis_2_ud | | Polyporaceae_ud |
|  |  | Diversisporaceae | Incertae_sedis_26 |  | Pseudeurotiaceae_ud |
|  |  | Valsaria | Hymenochaetales |  | Rhizopogon |
|  |  |  | Onygenaceae |  | Sporisorium |
|  |  |  | Sordariaceae |  | Trametes |
|  |  |  | Volutella |  | Truncatella |
|  |  |  | Williopsis |  | Tubeufiaceae_ud |
|  |  |  |  |  | Valsaceae |
|  |  |  |  |  | Incertae_sedis_25 |
|  |  |  |  |  | Ustilaginaceae |
|  |  |  |  |  | Entylomatales |
|  |  |  |  |  | Vuilleminia |
|  |  |  |  |  | Wallemia |
|  |  |  |  |  | Xylodon |

# Supplementary Figures

**Supplementary Figure 1.** Boxplot illustrating the differences in Shannon diversity measures of the fungal communities between vineyards **(a)**. Principal Coordinate Analysis (PCoA) based on Bray Curtis dissimilarity metrics, showing the distance in the fungal communities between vineyards **(b).**


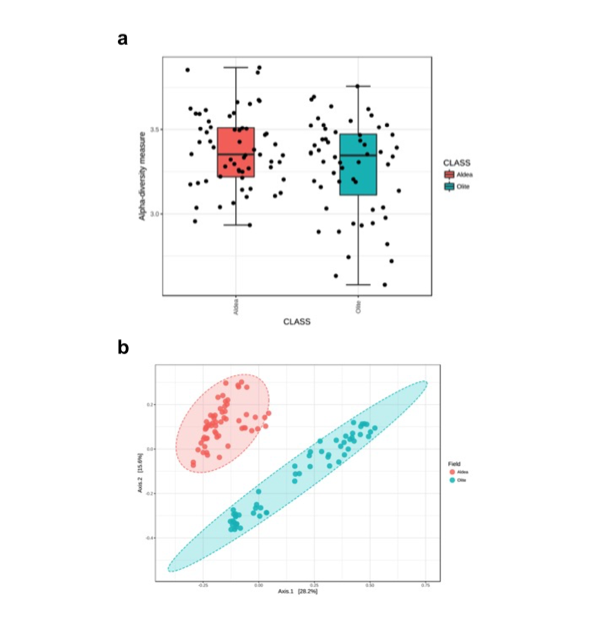


**Supplementary Figure 2.** Relative abundance of the most abundant families within the phyla Actinobacteria, Proteobacteria and Ascomycota in both vineyards representing OTUs showing more than 1% relative abundance of all reads and present in at least 2/3 of replicates. Families representing less than 1% of the total reads are grouped in ‘Others’.


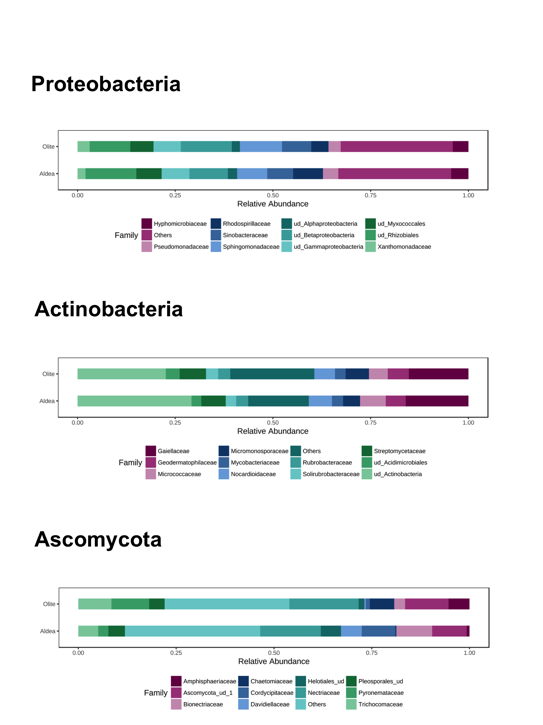


**Supplementary Figure 3**. Boxplot illustrating the differences in Chao1 richness measures of the bacterial communities between years of sampling in the grapevine rootstocks in Aldea (a) and Olite (b) vineyards. Principal Coordinate Analysis (PCoA) based on Bray Curtis dissimilarity metrics, showing the distance in the bacterial communities among grapevine rootstocks in Aldea (c) and Olite (d) vineyards.


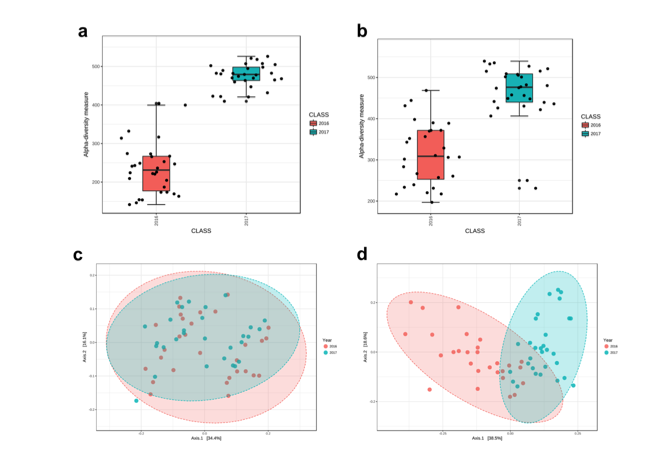


**Supplementary Figure 4.** Boxplot illustrating the differences in Chao1 richness measures of the fungal communities between years of sampling in the grapevine rootstocks in Aldea (a) and Olite (b) vineyards. Principal Coordinate Analysis (PCoA) based on Bray Curtis dissimilarity metrics, showing the distance in the fungal communities among grapevine rootstocks in Aldea (c) and Olite (d) vineyards.


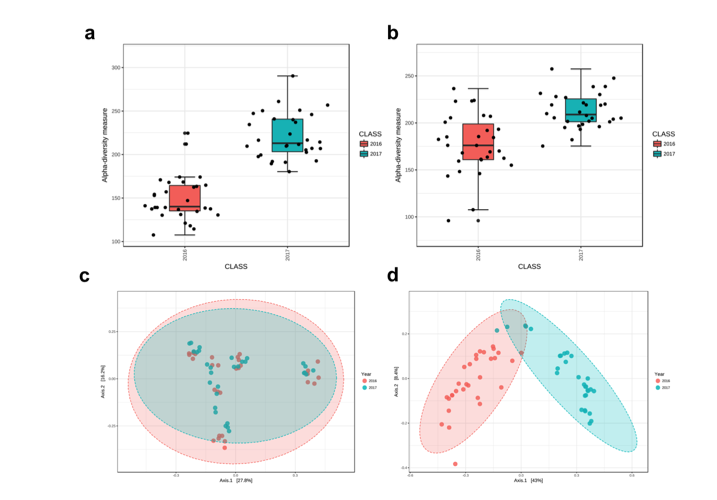


**Supplementary Figure 5.** Boxplot illustrating the differences in Shannon diversity measures of the fungal communities between sampling dates in the grapevine rootstocks in Aldea vineyard.


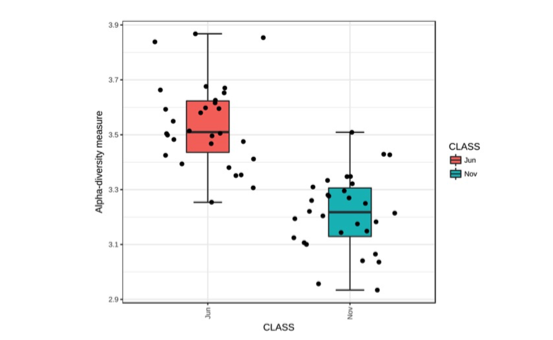


**Supplementary Figure 6.** Number of OTUs identified and DNA concentration of *Cylindrocarpon*-like asexual morphs for the five rootstocks analyzed in Aldea and Olite geographic regions in both years studied. Values are the mean of six replicates (3 samples per sampling time) and twelve replicates (3 samples per sampling time and 2 runs for each one) for qPCR and high-throughput amplicon sequencing analysis, respectively. Vertical bars represent the standard errors.


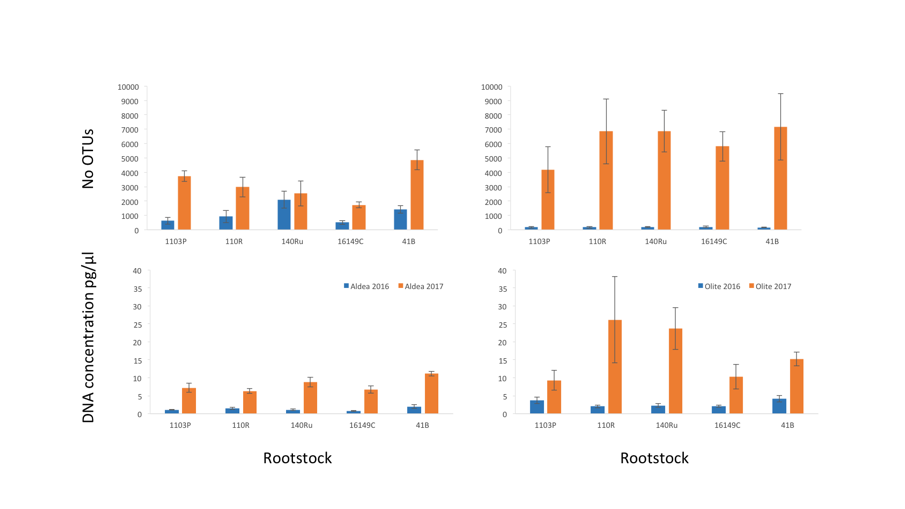


**Supplementary Figure 7.** The distribution of the number of OTUs and DNA concentration of *Cylindrocarpon*-like asexual morphs values are shown on the diagonal. The bivariate scatter plot with a fitted line is displayed on the bottom of the diagonal and the Spearman correlation value (*P*<0.05) is indicated on the top of the diagonal.


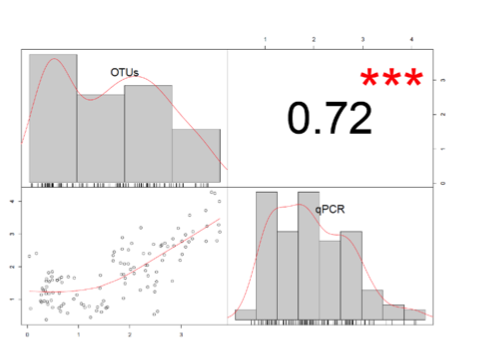

Supplement: Supplementary file 1 [file Data_Sheet_1.docx]
